# In Silico Prediction and Insights Into the Structural Basis of Drug Induced Nephrotoxicity

**DOI:** 10.3389/fphar.2021.793332

**Published:** 2022-01-05

**Authors:** Yinping Shi, Yuqing Hua, Baobao Wang, Ruiqiu Zhang, Xiao Li

**Affiliations:** ^1^ Shandong Medicine and Health Key Laboratory of Clinical Pharmacy, Department of Clinical Pharmacy, The First Affiliated Hospital of Shandong First Medical University and Shandong Provincial Qianfoshan Hospital, Jinan, China; ^2^ School of Pharmacy, Shandong First Medical University, Tai’an, China; ^3^ Department of Nephrology, The First Affiliated Hospital of Shandong First Medical University and Shandong Provincial Qianfoshan Hospital, Jinan, China; ^4^ Shandong Provincial Qianfoshan Hospital, Shandong University, Jinan, China

**Keywords:** drug induced nephrotoxicity, in silico prediction, consensus model, structural alert, web-server

## Abstract

Drug induced nephrotoxicity is a major clinical challenge, and it is always associated with higher costs for the pharmaceutical industry and due to detection during the late stages of drug development. It is desirable for improving the health outcomes for patients to distinguish nephrotoxic structures at an early stage of drug development. In this study, we focused on in silico prediction and insights into the structural basis of drug induced nephrotoxicity, based on reliable data on human nephrotoxicity. We collected 565 diverse chemical structures, including 287 nephrotoxic drugs on humans in the real world, and 278 non-nephrotoxic approved drugs. Several different machine learning and deep learning algorithms were employed for in silico model building. Then, a consensus model was developed based on three best individual models (RFR_QNPR, XGBOOST_QNPR, and CNF). The consensus model performed much better than individual models on internal validation and it achieved prediction accuracy of 86.24% external validation. The results of analysis of molecular properties differences between nephrotoxic and non-nephrotoxic structures indicated that several key molecular properties differ significantly, including molecular weight (MW), molecular polar surface area (MPSA), AlogP, number of hydrogen bond acceptors (nHBA), molecular solubility (LogS), the number of rotatable bonds (nRotB), and the number of aromatic rings (nAR). These molecular properties may be able to play an important part in the identification of nephrotoxic chemicals. Finally, 87 structural alerts for chemical nephrotoxicity were mined with f-score and positive rate analysis of substructures from Klekota-Roth fingerprint (KRFP). These structural alerts can well identify nephrotoxic drug structures in the data set. The in silico models and the structural alerts could be freely accessed via https://ochem.eu/article/140251 and http://www.sapredictor.cn, respectively. We hope the results should provide useful tools for early nephrotoxicity estimation in drug development.

## Introduction

Drug induced nephrotoxicity (DIN) can be defined as any renal injury caused directly or indirectly by medications (Sales and Foresto, 2020), which has been a major issue for patients and the pharmaceutical industry. The real world data (RWD) showed that incidence of drug induced nephrotoxicity to be approximately 14–26% in adult populations (Mehta et al., 2004; Uchino et al., 2005; Hoste et al., 2015). The syndrome always contributes to considerable morbidity, mortality, and high costs, and it can lead to the development of chronic kidney disease (CKD) or end-stage renal disease (ESRD). Nephrotoxicity has become an important concern in modern drug development. It is one of the major reasons for safety-related failures at all phases of drug development and even marketed drugs being restricted or withdrawn (Zhang et al., 2019).

The mechanisms of drug induced nephrotoxicity were very complex and may be different between various drug classes. Based on the histological component of the affected kidney, the drug induced nephrotoxicity should be categorized as three mechanisms, including proximal tubular injury and acute tubular necrosis (ATN), tubular obstruction by crystals or casts containing drugs and their metabolites, and interstitial nephritis induced by drugs and their metabolites (Nolin and Himmelfarb, 2010; Kwiatkowska et al., 2021). On the other hand, the mechanisms of drug induced nephrotoxicity can also be classified according to the mode of action of the drugs, including cytotoxicity (necrosis or apoptosis), immune injury, and ischemic injury. With the help of transdermal transport system, especially organic anion transporter 1 (OAT1), drugs were accumulated in proximal convoluted tubule epithelial cells. The cell necrosis or apoptosis would be caused when high concentration was reached, then cell necrosis or apoptosis would be caused (Sekine and Endou, 2009). It was reported that drugs can act on mitochondria and block production of adenosine triphosphate (ATP), resulting in cell necrosis or apoptosis (Gai et al., 2020). Nephrotoxic drugs can also increase superoxide free radical production and decrease antioxidant free radical production in epithelial cells, which can also lead to cell necrosis or apoptosis (Paller et al., 1984). Ferroptosis was a type of cell death characterized by iron overload and accumulation of toxic lipid peroxides (Li et al., 2020). In recent years, studies have shown that ferroptosis was closely related to drug induced nephrotoxicity, especially acute kidney injury, but the exact molecular biological mechanism has not been clarified, and this needs more research (Hu et al., 2019). The immune response caused by some drugs acting as antigens or haptens can result in inflammation of the blood vessels and tubules of the kidney, such as penicillin-induced interstitial nephritis (Spanou et al., 2006). This kind of immune damage was related to individual hypersensitivity to drugs, so there were significant individual differences. Besides, nephrotoxic drugs can cause renal ischemia and injury by reducing blood perfusion in renal tissues (Raza and Naureen, 2020). For example, nonsteroidal anti-inflammatory drugs (NSAIDs) were able to reduce renal blood flow by changing the resistance of glomerular entry and exit arteries, and then induce renal ischemic injury (Hörl, 2010). Unfortunately, there are few studies on the structural characteristics of nephrotoxic drugs.

The evaluation of the nephrotoxic potential of chemicals in early stage is quite important and useful for reducing the failure of drug development. The *in vivo* test for drug induced nephrotoxicity evaluation is always very complicated, costly, and time-consuming, and it is not suitable for screening a large number of chemicals, and especially for virtual structures. Besides, the experimental results are easily affected by various factors such as model animals and technology and environment. Compared with biological experimental methods, the use of computational toxicology for nephrotoxicity estimation of compounds has obvious advantages: (1) large quantities of compounds can be rapidly processed and predicted; (2) the toxicity of the compound can be predicted by computational models as long as the structure is known, even if the compound has not been synthesized; and (3) computational toxicological methods can also contribute to the study of the mechanisms. Consequently, it should make a lot of sense to develop fast and accurate computational tools to estimate the risk of nephrotoxicity. Over the past decades, many computational models have been developed for toxicity prediction, but only a few models reported related to kidney injury or urinary tract toxicity, and due to the variety and complexity of the symptoms and mechanisms. Lei et al. summarized the reported models related to urinary tract toxicity until 2017 (Lei et al., 2017). Most of them were established based on biomarker descriptors, and only five models were based on theoretical descriptors with the datasets of drugs in various development phases. In Lei et al.’s study, they developed a series of qualitative and quantitative structure activity relationship (QSAR) models for urinary tract toxicity prediction using 258 compounds, the best regression model reached q^2^_ext_ of 0.845 for the test set, and the best classification model gave global accuracy of 90.77% for the test set. Zhang et al. developed an *in silico* prediction model for chemical induced urinary tract toxicity using naïve Bayes classifier based on mouse intraperitoneal data set (Zhang et al., 2019). The model provided 84.2% overall accuracy for the external test set. They also obtained several important molecular descriptors and fragments. More recently, Sun et al. developed QSAR models for screening nephrotoxicity of the ingredients in TCMs based on natural product or mixed dataset (Sun et al., 2019). The models performed well on external validation with 30 ingredients in the TCMs. The published models related to nephrotoxicity always provided high statistical performance. However, the structural characteristics of nephrotoxic and non-nephrotoxic drugs were rarely analyzed in these studies, and the usefulness of most published models was restricted because of poor availability. Besides, it should be more useful to develop the models based on real world data with human nephrotoxicity, and due to the species specificity in drug toxicity between rodent animals and human beings.

In the present study, we focused on the *in silico* prediction and insights into the structural basis of drug induced nephrotoxicity based on the medications causing human nephrotoxicity in the real world.

## Materials and Methods

### Data Source and Preparation

In this study, only medications with human nephrotoxicity data were included. The nephrotoxic structures were extracted from the Side Effect Resource (SIDER) database (Kuhn et al., 2016). SIDER is a widely used database of adverse drug reactions (ADRs), which contained the information on approved drugs and the ADRs on humans. Herein, we retrieved the entire SIDER database and collected those drugs with nephrotoxicity related ADRs with frequency ≥0.1% in the real world. The corresponding structures of included chemicals were downloaded in smiles format from the PubChem database (Kim et al., 2016). The non-nephrotoxic structures were extracted from Zhang’s work (Zhang et al., 2019). They built the negative drug dataset with the drugs without nephrotoxicity from the SIDER database. The included structures were carefully prepared as follows: (1) removing duplicate substances; (2) keep only the main ingredients in mixtures; and (3) salts were converted to their parent forms.

### Principal Component Analysis for the Definition of the Chemical Space

The sufficiently structural diversity was a key issue for global QSAR models to ensure a reasonable predictive accuracy (Ancuceanu et al., 2019). Principal component analysis (PCA) is a well-known technique for reducing the dimensionality and increasing interpretability, which can solve an eigenvector problem by creating new uncorrelated variables that successively maximize variance (Jolliffe and Cadima, 2016). In this study, the chemical space of data sets was analyzed with the first two principal components of CDK (Chemistry Development Kit) Descriptors. The PCA was performed using SPSS Statistics 26.

### Algorithms For Model Building

The model building was performed on the online chemical database and modeling environment (OCHEM), which is a user friendly web-based platform for automatic and simple QSAR modeling (Sushko et al., 2011). OCHEM supports the typical steps of QSAR modeling, and the models can be published and publicly used on the web (Oprisiu et al., 2013; Cui et al., 2019; Pawar et al., 2019; Cui et al., 2021; Hua et al., 2021; Huang et al., 2021; Ta et al., 2021). Among the many state-of-the-art modeling methods available on OCHEM, we applied five widely used traditional machine learning (ML) approaches and five different deep learning (DL) algorithms. As an application of artificial intelligence (AI), ML has been an effective tool for modeling in computational toxicology. Five highly effective and robust ML approaches were used in this study, including associative neural network (ASNN) (Tetko, 2009), support vector machine (SVM) (Chang and Lin, 2011), C4.5 decision tree (WEKA J48) (Hall et al., 2009), random forest (RFR) (Breiman, 2001), and extreme gradient boosting (XGBoost) (Chen and Guestrin, 2016). DL is an extension of machine learning, and its concept came from the research of artificial neural networks. In this study, we used five different DL approaches, including convolutional neural network fingerprint (CNF) (Tetko et al., 2019), transformer convolutional neural network (TRANSNN) (Karpov et al., 2020), TEXTCNN algorithm available from DeepChem (TEXTCNN) (Wu et al., 2017), Graph Isomorphism Network (GIN) (Capela et al., 2019), and edge attention based multi-relational graph convolutional networks (EAGCNG) (Shang et al., 2018). The detailed descriptions of these ML and DL approaches can be found in the corresponding literature.

The individual parameters for each model algorithm were optimized automatically by the method itself in an inner loop of cross-validation. For instance, the SVM algorithm used libSVM (Chang and Lin, 2011) on OCHEM. There were two important configurable options for this method, including SVM type (ε-SVR and μ-SVR, etc.) and the kernel type (linear, polynomial, radial basis function, sigmoid, etc.). In the OCHEM workflow, classic ε-SVR and radial basis function kernels were used. The other SVM parameters, namely cost C and width of the RBF kernel (γ, g), were optimized using default grid search, and this was performed according to the LibSVM manual (Tetko et al., 2013).

### Molecular Description

For the machine learning modeling, the molecular descriptors were served as the input of drug structures. We calculated eight different descriptor packages, including Chemaxon descriptors (Chemaxon, 499 descriptors), Fragmentor, GSFrag descriptors (GSFrag, 1,138 descriptors), MORDRED descriptors (MORDRED, 1826 descriptors), PyDescriptor (1,624 descriptors), QNPR descriptors (QNPR), RDKit descriptors (RDKit), and alvaDesc descriptors (5,666 descriptors). The details of these descriptor packages can be learned via http://docs.ochem.eu//display/MAN/Molecular+descriptors.html. The descriptors were filtered with pairwise de-correlation method before the model building. There was no selection bias, since the unsupervised filtering was totally independent.

For the deep learning models, the SMILES string of each compound was served as the input without descriptors.

### Consensus Modeling

Consensus modeling is to unify the prediction of unknown samples by multiple individual models to achieve a unified result, and then improve the prediction of the model. By averaging the prediction of individual models, noise can be reduced, thus, and the consensus model can provide better predictive power than most individual models alone (Tapia Garcı’a et al., 2012). Herein, the consensus model was built with simple average of predictions from the best performed individual models.

### Applicability Domain Assessment

For QSAR models, it is important to estimate the applicability domain (AD) to determine whether the test compound is suitable for the specific model. In this study, we determined the AD with distance to model (DM) in OCHEM proposed by Sushko (Sushko et al., 2011). The DM assesses the distance from the target compound to the model. The larger DM means the lower applicability for the target compound.

### Analysis of Molecular Properties Differences Between Nephrotoxic and Non-nephrotoxic Structures

The molecular properties of compounds always can make a significant difference in the toxicity. In this study, the analysis of differences of molecular properties between nephrotoxic and non-nephrotoxic structures was performed, in order to investigate the relevance of these molecular properties with drug induced nephrotoxicity. Several commonly used physical-chemical properties which have been widely adopted in the analysis for other endpoints were calculated and analyzed, including molecular weight (MW), molecular polar surface area (MPSA), AlogP, molecular solubility (LogS), the number of hydrogen bond acceptors (nHBA) and donors (nHBD), the number of rotatable bonds (nRotB), and the number of aromatic rings (nAR). These properties are relative to the molecular size, lipophilicity and solubility, and hydrogen bonding ability and complexity, respectively. The comparison between groups was tested by T-test, and the *p* value <0.05 was considered to indicate statistical significance. The molecular properties were calculated with PaDEL-Descriptor package (Yap, 2011).

### Identification of Structural Alerts Responsible for Nephrotoxicity

Structural alert (SA), or privileged substructure, was defined as the substructure which can cause the chemicals to become toxic. It has been well accepted in toxicity assessment, because of the direct derivation from mechanistic knowledge. SAs have been commonly used for toxicity assessment of many different endpoints (Claesson and Minidis, 2018; Yang et al., 2018; Wedlake et al., 2020). In this study, we identified the structural alerts for nephrotoxicity by calculating f-score and positive rate of each fragment from Klekota-Roth fingerprint (KRFP, 4,860 bits). The specific substructure should be regarded as a SA if presented more frequently in nephrotoxic drugs than non-nephrotoxic drugs. The positive rate (PR) of a substructure was defined as below:

$$PR=\frac{N_{fragment\_positive}}{N_{fragment}}$$ (1)

where N_*fragment_positive*_ was the number of nephrotoxic drugs containing the fragment, and N_*fragment*_ was the total number of drugs containing the fragment.

### Validation and Evaluation of Models

All the ML and DL models were first internally validated with 5-fold cross validation, and the best performed models were further validated with the external validation set. The structural alerts were assessed with the whole dataset. The classifiers and SAs were evaluated based on the counts of true positives (TP), false positives (FP), true negatives (TN), and false negatives (FN). Several statistical parameters were calculated to evaluate the classifiers, including the total accuracy (Q), sensitivity (SE), specificity (SP), enrichment factor (EF), and Matthews correlation coefficient (MCC), which were calculated with Eqs (2–6).

$$Q=\frac{TP+TN}{TP+FN+TN+FP}$$ (2)

$$SE=\frac{TP}{TP+FN}$$ (3)

$$SP=\frac{TN}{TN+FP}$$ (4)

$$EF=\frac{TN/\left(TN+FN\right)}{\left(TN+FP\right)/\left(TP+FN+TN+FP\right)}$$ (5)

$$MCC=\frac{TP*TN-FP*FN}{\sqrt{\left(TP+FP\right)\left(TP+FN\right)\left(TN+FP\right)\left(TN+FN\right)}}$$ (6)

Additionally, the receiver operating characteristic (ROC) curve was also plotted for the QSAR models, and the values of area under the ROC curve (AUC) were provided, too.

## Results and Discussion

### Data Set Analysis

In the study, 565 diverse structures were kept after preparation, including 287 nephrotoxic drugs and 278 non-nephrotoxic drugs. As shown in Table 1, the whole data set was randomly divided into a training set with 456 chemicals (232 nephrotoxic and 224 non-nephrotoxic) and an external validation set with 109 chemicals (55 nephrotoxic and 54 non-nephrotoxic). The structures of the drugs can be found in Supplementary Table S1.

**TABLE 1:** The number of structures in the data set.

	Nephrotoxic structures	Non-nephrotoxic structures	Total
Training set	232	224	456
Validation set	55	54	109
Total	287	278	565

**FIGURE 1:**
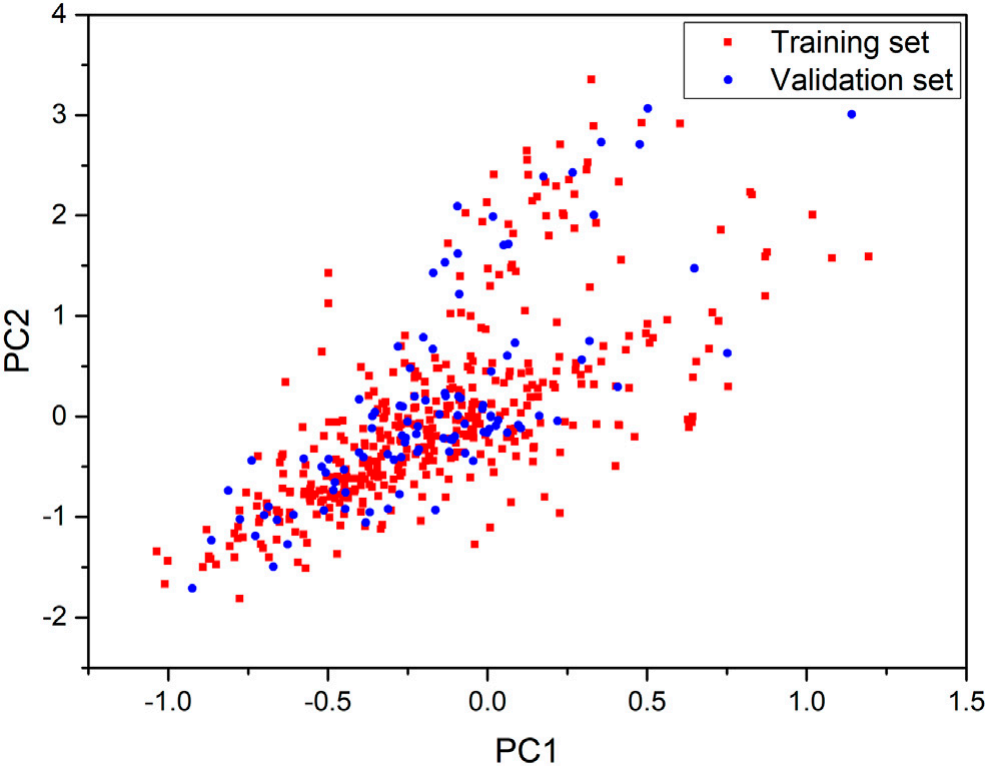
Chemical space defined by the first two principal components of CDK descriptors. Red squares stand for the training set, blue circles stand for the validation set.

The diversity of structures is a crucial factor for the applicability of global models. Thus, we performed the principal component analysis (PCA) based on CDK descriptors to analyze the chemical space of the included compounds. PCA can simplify the complexity in high-dimensional data while retaining trends and patterns by transforming the data into fewer dimensions, which act as summaries of features (Ringnér, 2008). The first two principal components were kept and used for the definition of the chemical space of compound in the training and validation sets in this study. As shown in Figure 1, the distribution scatter diagram illustrated that the data sets shared a similar chemical space. In addition, the Tanimoto similarity index (TSI) was also calculated based on the ECFP-4 fingerprint to evaluate similarities among the structures. The average value of the entire data set was 0.13, which indicated an evidently chemical diversity of the entire data set.

### Results of ML and DL Models

Combined with five different ML algorithms and eight molecule descriptor packages, 40 ML models were developed. Meanwhile, five DL models were developed with different algorithms using chemical SMILES-string as input. The performances of ML and DL models on 5-fold cross validation were shown in Table 2. The prediction accuracy (Q) of the models ranged from 58.54 to 73.90%. Among them, three models performed much better on 5-fold cross validation than others, including a DL model (CNF) and two ML models (XGBoost_QNPR and RFR_QNPR). These models all provide good predictive ability with Q value ≥ 70.00% and AUC value ≥0.80.

**TABLE 2:** Performances of models on 5-fold cross-validation.

Model	Q (%)	SE (%)	SP (%)	EF	MCC	AUC
XGBOOST_QNPR	72.81	71.98	73.66	1.46	0.46	0.80
WEKA_J48_QNPR	67.11	69.83	64.29	1.37	0.34	0.67
RFR_QNPR	72.15	71.98	72.32	1.45	0.44	0.80
libSVM_QNPR	68.20	66.81	69.64	1.36	0.36	0.68
ASNN_QNPR	69.30	68.97	69.64	1.39	0.39	0.75
XGBOOST_PyDescriptor	63.82	62.07	65.63	1.27	0.28	0.70
WEKA_J48_PyDescriptor	60.09	60.78	59.38	1.21	0.20	0.60
RFR_PyDescriptor	68.86	69.83	67.86	1.39	0.38	0.74
libSVM_PyDescriptor	63.38	58.19	68.75	1.25	0.27	0.63
ASNN_PyDescriptor	63.38	62.07	64.73	1.27	0.27	0.68
XGBOOST_MORDRED	64.04	65.09	62.95	1.29	0.28	0.69
WEKA_J48_MORDRED	67.32	70.69	63.84	1.38	0.35	0.67
RFR_MORDRED	67.98	67.67	68.30	1.37	0.36	0.75
libSVM_MORDRED	67.54	66.81	68.30	1.35	0.35	0.68
ASNN_MORDRED	66.01	65.09	66.96	1.32	0.32	0.71
XGBOOST_GSFrag	63.86	64.32	63.39	1.28	0.28	0.68
WEKA_J48_GSFrag	62.53	60.79	64.29	1.24	0.25	0.63
RFR_GSFrag	63.86	61.23	66.52	1.27	0.28	0.70
libSVM_GSFrag	64.75	57.27	72.32	1.26	0.30	0.65
ASNN_GSFrag	63.41	57.27	69.64	1.24	0.27	0.67
XGBOOST_Fragmentor	63.16	62.07	64.29	1.26	0.26	0.70
WEKA_J48_Fragmentor	58.77	55.17	62.50	1.17	0.18	0.59
RFR_Fragmentor	67.54	65.95	69.20	1.35	0.35	0.73
libSVM_Fragmentor	67.76	66.38	69.20	1.35	0.36	0.68
ASNN_Fragmentor	64.47	63.36	65.63	1.29	0.29	0.69
XGBOOST_ECFP4	63.82	63.36	64.29	1.28	0.28	0.70
WEKA_J48_ECFP4	59.21	56.03	62.50	1.18	0.19	0.59
RFR_ECFP4	66.45	63.79	69.20	1.32	0.33	0.73
libSVM_ECFP4	65.79	63.79	67.86	1.31	0.32	0.66
ASNN_ECFP4	65.57	64.66	66.52	1.31	0.31	0.67
XGBOOST_Chemaxon	62.97	64.63	61.26	1.27	0.26	0.67
WEKA_J48_Chemaxon	61.64	57.64	65.77	1.22	0.23	0.62
RFR_Chemaxon	64.30	61.57	67.12	1.28	0.29	0.71
libSVM_Chemaxon	64.30	58.52	70.27	1.26	0.29	0.64
ASNN_Chemaxon	64.97	65.07	64.86	1.31	0.30	0.70
XGBOOST_alvaDesc	64.04	64.22	63.84	1.29	0.28	0.69
WEKA_J48_alvaDesc	63.38	64.66	62.05	1.28	0.27	0.63
RFR_alvaDesc	70.18	68.97	71.43	1.40	0.40	0.75
libSVM_alvaDesc	65.13	68.53	61.61	1.33	0.30	0.65
ASNN_alvaDesc	70.61	68.97	72.32	1.41	0.41	0.73
CNF	73.90	69.83	78.13	1.45	0.48	0.81
TRANSNNI	69.45	65.80	73.21	1.37	0.39	0.74
GNN GIN	67.11	67.24	66.96	1.35	0.34	0.74
EAGCNG	58.54	52.42	64.73	1.15	0.17	0.62
DEEPCHEM	70.42	53.39	80.83	1.19	0.36	0.74
Consensus	75.88	72.84	79.02	1.50	0.52	0.83

The consensus approach has been proved able to improve the accuracy of models. In this study, a consensus model was developed based on the three best models. The consensus model performed much better than the individual models. As shown in Table 2, it provided the Q value of 75.88% and the AUC value 0.83; the values of SP, SE, EF, and MCC were 72.84, 79.01, 1.50, and 0.52%, respectively.

### External Validation of Models

**FIGURE 2:**
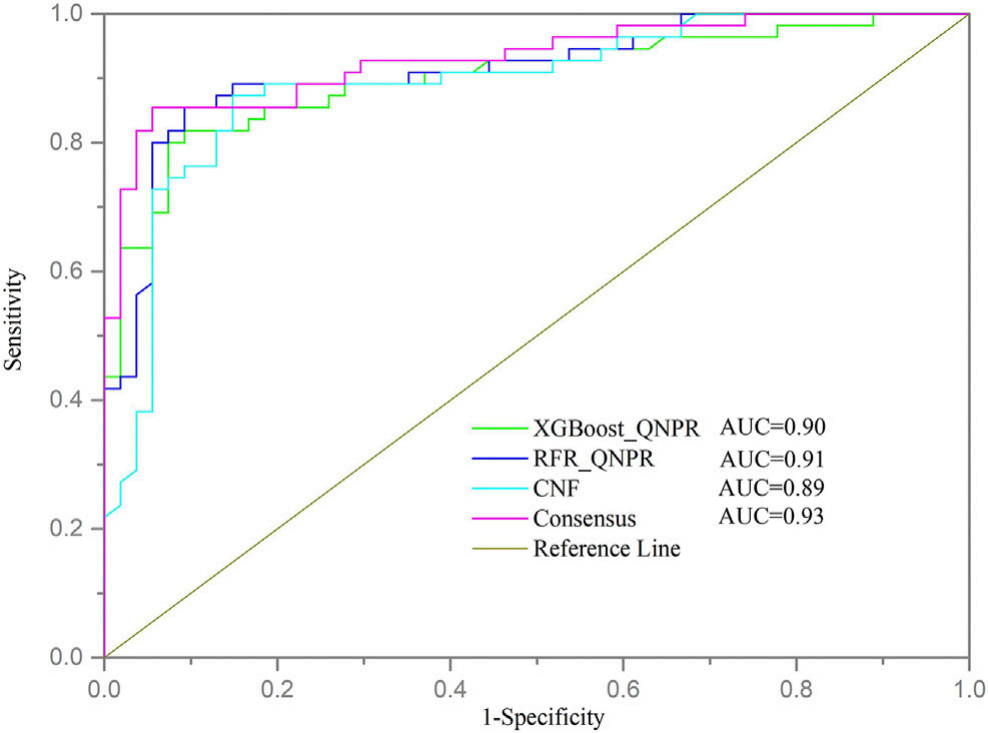
ROC curve of models on external validation. Each color line represents a model.

Due to the complete independence from the model training, the external validation set could be well used for evaluating the predictive ability of models objectively. As shown in Table 3; Figure 2, the best performed individual models also achieved good predictive results on external validation. The model developed with RFR algorithm and QNPR descriptors performed best with Q value 87.16% and AUC value 0.91. The DL model developed with CNF algorithm also provided good predictive ability, with Q value 83.49%, and AUC value 0.89. The consensus model did not perform better than RFR_QNPR model on most of the statistical parameters, except for AUC value (0.93). It provided a Q value of 86.24% and MCC value of 0.82 on external validation, and the values of SE, SP, and EF were 85.45, 87.04, and 1.72%, respectively.

**TABLE 3:** Performances of models on external validation.

Model	Q (%)	SE (%)	SP (%)	EF	MCC	AUC
RFR_QNPR	87.16	87.27	87.04	1.76	0.74	0.91
XGBOOST_QNPR	83.49	85.45	81.48	1.71	0.67	0.90
CNF	83.49	80.00	87.04	1.64	0.67	0.89
Consensus	86.24	85.45	87.04	1.72	0.72	0.93

The results of ML model building and validation suggested that ML models developed with the QNPR descriptor performed much better than others. The QNPR descriptors were derived directly from the chemical SMILES strings. For each structure either canonical SMILES or IUPAC name would be split into fragments of a specified length determined by the configuration (Thormann et al., 2007). For the different ML algorithms, XGBoost and RFR performed better than others. XGBoost is a scalable end-to-end tree boosting system, which has been widely used in many application scenarios. It supports multi-thread computation and uses regularization enhancement technology to reduce over-fitting, so as to ensure the robustness of the model. Meanwhile, it has the advantages of flexibility, fast calculation speed, and good robustness. Therefore, XGBoost is not easy to be disturbed by outliers. It can achieve state-of-the-art results on many machine learning challenges (Chen and Guestrin, 2016). RFR is an ensemble learning method by constructing a multitude of decision trees (DT) at training time (Breiman, 2001). It can help to improve the accuracy by reducing overfitting in decision trees and automating missing values present in the data. CNF contributed to the best DL model. CNF is one of the state-of-the-art deep learning methods, which is based on ideas of text processing. It could achieve the high prediction accuracy due to the augmentation technique employed during both training and inference steps.

The DL model in our study did not show significant better predictive power compared with the ML model. However, considering that the DL models did not require additional molecular description, as long as SMILES are provided, the DL algorithms still showed obvious advantages over ML algorithms.

### The Differences of Molecular Properties Between Nephrotoxic and Non-nephrotoxic Drugs

**FIGURE 3:**
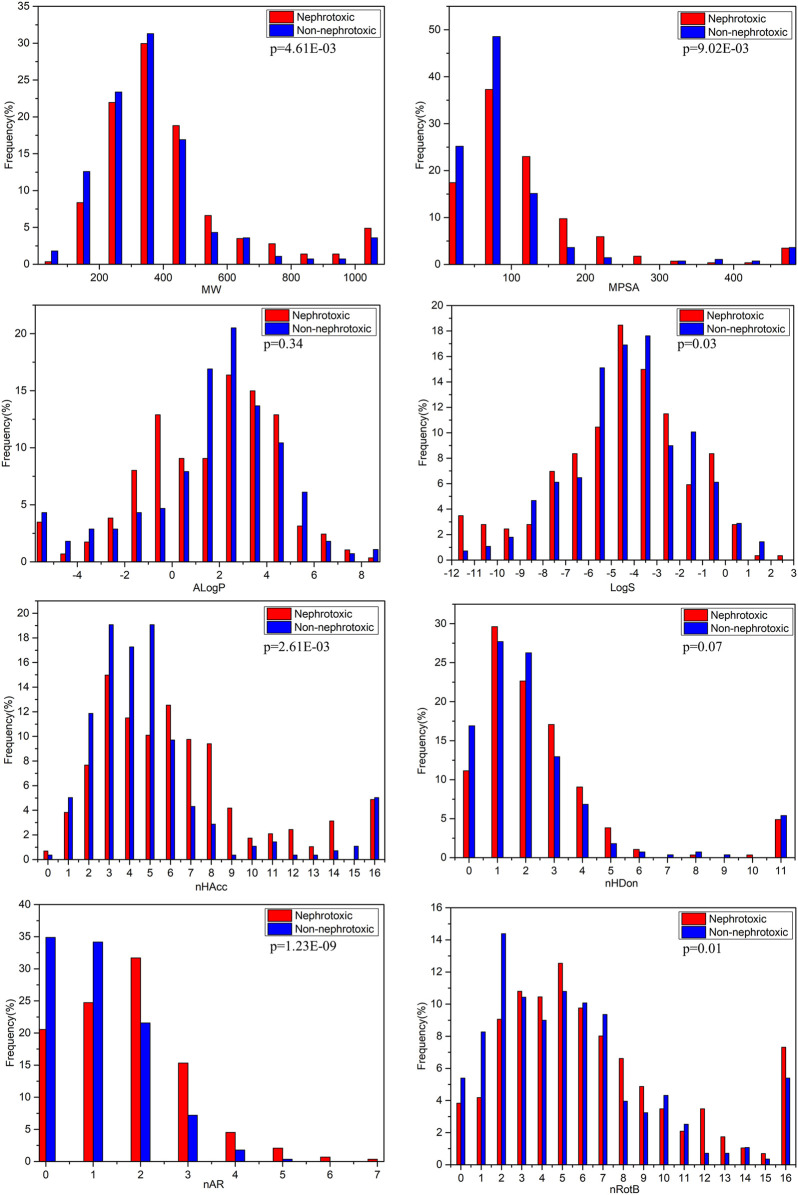
Distributions of the commonly molecular properties for nephrotoxic and non-nephrotoxic drugs.

The molecular physical-chemical properties could provide useful information for biological activities. Herein, we analyzed the differences of several commonly used physical-chemical properties between nephrotoxic and non-nephrotoxic drugs. The distributions of these descriptors for nephrotoxic and non-nephrotoxic drugs can be seen in Figure 3.

The characteristics MW and MPSA can simply assess the size and complexity of compounds. For the entire data set in the study, the values of MW were distributed between 59.04 and 4,491.88, with a mean of 416.86. The mean value was 459.71 for nephrotoxic drugs and 372.63 for non-nephrotoxic drugs. The difference between the mean MW of nephrotoxic and non-nephrotoxic drugs was not significantly different (*p* < 0.01). The values of MPSA were distributed from 0 to 1902.88, with a mean of 119.80 for the entire dataset. The mean value was 137.52 for nephrotoxic drugs and 101.51 for non-nephrotoxic drugs (*p* < 0.01). These results suggested that it may be significantly different in structure size and polar surface area between nephrotoxic and non-nephrotoxic compounds.

The AlogP is commonly used to represent the lipophilicity of compounds. For the entire data set, the values of AlogP ranged from −32.81 to 10.25, with a mean of 1.48. The mean value of AlogP was 1.34 for nephrotoxic chemicals and 1.64 for non-nephrotoxic drugs. As can be seen in Figure 3, the distributions were not significantly different between nephrotoxic and non-nephrotoxic drugs with *p*-value 0.34. The result indicated that chemical lipophilicity may be weakly correlated with drug induced nephrotoxicity.

Chemical hydrogen bonding ability also is an important character for its activity and toxicity, and it was usually in terms of nHBA and nHBD. In this data set, mean values of nHBA for nephrotoxic and non-nephrotoxic drugs were 7.06 and 5.41, respectively, and the difference was significant (*p* < 0.01). Meanwhile, the nephrotoxic and non-nephrotoxic drugs had mean values of nHBD with 3.29 and 2.47, respectively, and the difference is not significant (*p* = 0.07). The data indicated that nHBA was obviously associated with drug induced nephrotoxicity while nHBD was not.

LogS is an estimation of molecular solubility in water. For the entire data set, the values of LogS were distributed from −35.86 to 2.45, and the mean value was −4.52. For nephrotoxic structures, the mean value of LogS was −4.81, and it is −4.21 for non-nephrotoxic drugs. The difference also proved statistical significance (*p* = 0.03). This result demonstrated that there was difference of molecular solubility between nephrotoxic and non-nephrotoxic drugs.

As shown in Figure 3, drug induced nephrotoxicity was also obviously associated with nRotB (the mean values were 8.05 for nephrotoxic chemicals, and 5.78 for non-nephrotoxic drugs, and with *p*-value 0.01) and nAR (the mean values were 1.70 for nephrotoxic chemicals and 1.08 for non-nephrotoxic drugs, with significant difference (*p* < 0.01).

Through the analysis of molecular properties, several physical-chemical properties have obvious differentiating effect on drug induced nephrotoxicity. Nevertheless, nephrotoxicity is a complex endpoint yet. It’s not easy to explain the mechanism of drug induced nephrotoxicity with individual simple chemical descriptors.

### Structural Alerts Responsible for Nephrotoxicity

The structural alerts (SA) responsible for nephrotoxicity were identified using f-score and positive rate analysis of each fragment from KRFP fingerprint. Only the fragments existing in 6 or more drugs were kept. The fragments with f-score ≥0.005 and positive rate ≥0.75 were identified. Finally, 87 representative fragments were filtered and listed in Supplementary Table S2. Among them, 16 substructures presented in nephrotoxic active chemicals only, which covered 76 nephrotoxic drugs. Details of each fragment and the representative structures were shown in Table 4.

**TABLE 4:** Structural alerts only presented in nephrotoxic drugs.

ID	Bit	SMARTS	Positive	Negative	Representative structure
1	KR413	[!#1][CH2][CH2]c1[cH][cH][cH][cH][cH]1	9	0	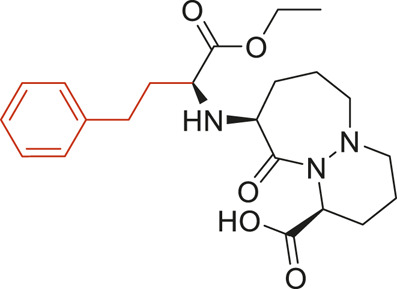
2	KR848	[!#1][NH]C(=O)[CH]([CH3])[NH]C(=O)[!#1]	7	0	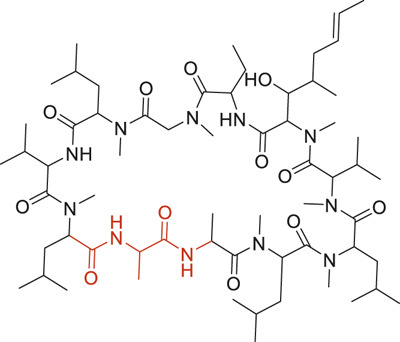
3	KR1798	[!#1]c1[cH][cH]c(F)[cH][cH]1	8	0	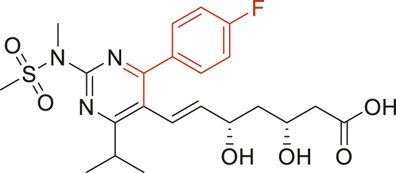
4	KR2444	[!#1]N1[CH2][CH2]N([CH3])[CH2][CH2]1	6	0	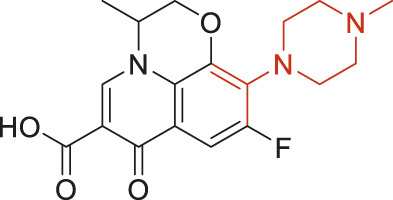
5	KR3206	c1nc2ccccc2[nH]1	7	0	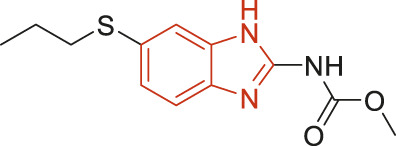
6	KR3280	CC(=O)c1ccc(N)cc1	8	0	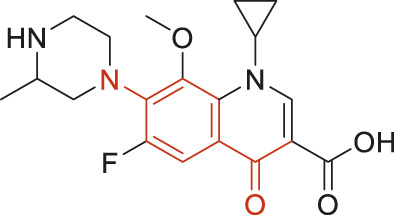
7	KR3540	Cc1ccc(cc1)c2ccc​cc2	7	0	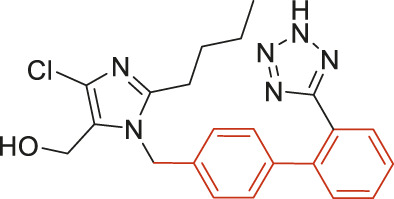
8	KR3548	Cc1ccc(F)cc1	8	0	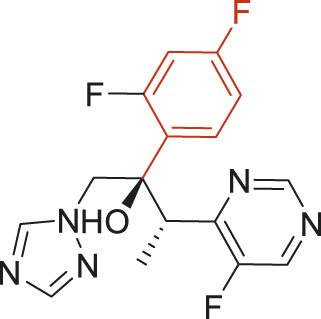
9	KR3586	Cc1cccc(F)c1	12	0	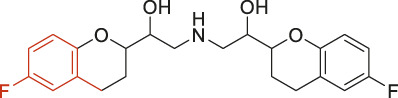
10	KR4029	CS(c1nc2ccccc2[nH]1)	6	0	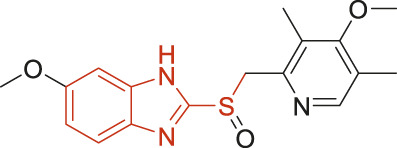
11	KR4064	Fc1cccc(C=O)c1	8	0	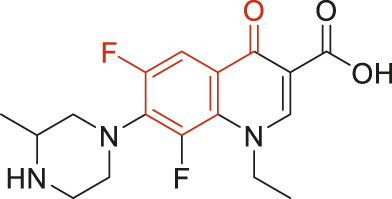
12	KR4065	Fc1cccc(F)c1	8	0	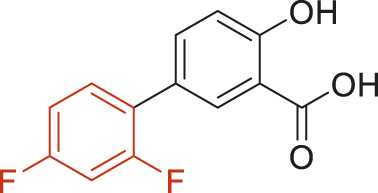
13	KR4081	N#Cc1ccccc1	6	0	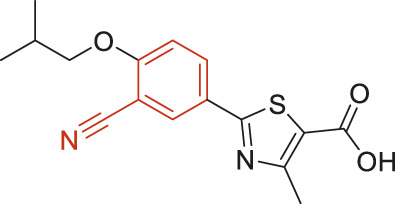
14	KR4252	Nc1ccc(F)cc1	11	0	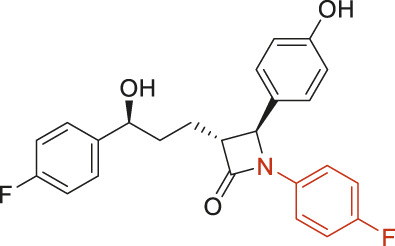
15	KR4556	O=CNCCCCNC = O	8	0	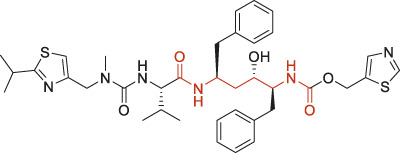
16	KR4651	OC(=O)C1CCCN1	8	0	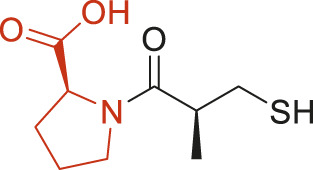

We analyzed the structural characteristics of drugs in the entire data set, and 75.04% structures (424/565) were correctly classified. Among the 290 drug structures contained at least one identified substructure, 218 structures were true nephrotoxic. The classification accuracy was 75.17%. For the drug structures that did not contain any identified substructure, 206 of 275 drugs (74.91%) were true non-nephrotoxic. The frequencies of these substructures were much higher in nephrotoxic drugs than non-nephrotoxic drugs, and showed a good ability to distinguish nephrotoxic drugs in the whole data set. To a certain degree, these fragments could be considered as the structural alerts responsible for nephrotoxicity. If a structure contains one or more structural alerts, it is more likely to be nephrotoxic than non-nephrotoxic.

Fluorine is widely used in medicinal chemistry to improve a molecule’s potency and permeability. However, the metabolism of fluorinated compounds may produce fluoride and other toxic metabolites (Pan, 2019). As shown in Table 4, several selected structural alerts contained phenyl fluoride (Nos 3, 8, 9, 11, 12, and 14). Since the kidney is a main target organ of mammalian fluoride systemic exposure and renal toxicity can occur after acute and chronic fluoride intoxication (Quadri et al., 2016). The kidney plays a vital role in fluoride metabolism, since 50–80% of fluoride is removed via urinary excretion. Fluorinated compounds can be toxic to the kidneys in a number of ways. It has widely been reported that fluorinated compounds can increase the generation of reactive oxygen species (ROS) and free radicals, cause extensive oxidative stress and excessive lipid peroxidation, and reduce antioxidant enzyme activities *in vivo* or *in vitro* (Quadri et al., 2016). Thus, numerous renal structural, ultrastructural, and functional may be changed after receiving increased amounts of fluorinated compounds exposure. Certainly, this is not meant to sound the alarm on all fluorinated compounds, we suggest raising the awareness of common drug instability and metabolism issues leading to defluorination, as well as the resulting reactive/toxic metabolite (Pan, 2019). Polyamines and their derivatives (Nos 2 and 15) widely existed in aminoglycosides and the other nephrotoxic drugs. Polyamines catabolism can be stimulated through oxidation, which will lead to the generation of ROS and low abundance of free polyamines with antioxidant capacity (Pegg, 2013; Murray Stewart et al., 2018). Consequently, this process is associated with a number of pathologies, including cancer, neurological disorders, and kidney dysfunction. Recent studies have also suggested a role for polyamines derivatives in the p53-mediated ferroptotic response to ROS stress. It is an iron-dependent and nonapoptotic mode of cell death and has been associated with drug-induced nephrotoxicity recently (Ou et al., 2016; Murray Stewart et al., 2018). Benzimidazoles derivatives were always proposed as new bioreductive prodrugs with the potential anticancer activity, due to their effect on the DNA destruction and growth inhibition into selected tumor cell lines (Błaszczak-Świątkiewicz et al., 2014). Besides, benzimidazoles derivatives also can induce concurrent apoptotic and pyroptotic cell death (Ren et al., 2021). However, when these processes take place in kidney cells, kidney damage can occur. Toluene-contained structures (Nos 1 and 7) were also selected as structural alerts for drug induced rhabdomyolysis (Cui et al., 2019), which always associated to oliguric renal failure. In the setting of toluene intoxication, electrolyte disturbances may play important roles on causing rhabdomyolysis (Camara-Lemarroy et al., 2015). Toluene has also been associated with direct induction of acute tubular necrosis and acute oliguric renal failure. Among the SAs we identified, there were several fragments that also could cause the formation of ROS (Nos 6 and 16, etc.), which may contribute to the nephrotoxicity.

In the present study, the SAs were identified with the f-score and frequency analysis of defined substructures. These methods have been widely used for the SA discovery for many other endpoints. In fact, these methods have some shortcomings. They are not able to characterize the spatial arrangement of identified substructures, and they cannot make a good distinction when more than one SA presented in the same structure. In spite of this, these structural alerts were able to well distinguish chemical structures with renal toxicity, and they can help to understand the specific fragments which lead to nephrotoxicity. Therefore, these SAs should be severed as a useful tool to visually evaluate the nephrotoxicity of chemicals.

### Availability of QSAR Models and Structural Alerts

For ease of use, the QSAR models were made available at OCHEM. The consensus model could be accessed via https://ochem.eu/article/140251. The three best individual models (RFR_QNPR, XGBOOST_QNPR, and CNF) were also available with the corresponding model IDs. Users can predict chemical nephrotoxicity by using the “Apply the model to new compounds” link. In addition, the data sets for modeling could be downloaded by using the “Export this basket” link.

The structural alerts responsible for nephrotoxicity have been integrated as part of our Web server SApredictor, which is a structural alert based expert system for drug toxicity prediction and freely available at http://www.sapredictor.cn. With the help of SApredictor, people can quickly evaluate whether the query chemicals are nephrotoxic, and the specific structural fragments that lead to the nephrotoxicity of the compounds will be intuitively shown to provide valuable reference for the modification of the structures.

## Conclusion

In this study, we collected 287 drug structures which were proved nephrotoxic on humans in the real world. A comparable amount of non-nephrotoxic structures was also extracted from approved drugs. Then, *in silci*o models were developed using OCHEM tools. A total of 40 ML models were developed using 5 different machine learning algorithms along with 8 descriptor packages. Besides, 5 DL models were also developed using different deep learning methods. Among them, two ML models (RFR_QNPR and XGBoost_QNPR), and one DL models (CNF) provided best predictive ability. A consensus model was developed based on them, which performed much better on internal validation, and provided good predictive ability on external validation. The consensus model and the best individual models were freely available at https://ochem.eu/article/140251. Moreover, the differences of several commonly used physical-chemical properties between nephrotoxic and non-nephrotoxic drugs were investigated. The results indicated that several key molecular properties differ significantly between nephrotoxic and non-nephrotoxic structures, including molecular weight (MW), molecular polar surface area (MPSA), AlogP, number of hydrogen bond acceptors (nHBA), molecular solubility (LogS), the number of rotatable bonds (nRotB), and the number of aromatic rings (nAR). Thus, these molecular descriptors may be associated to drug-induced nephrotoxicity and could play an important role in the identification of nephrotoxic chemicals. Finally, we identified the structural alerts responsible for nephrotoxicity using f-score and positive rate analysis. There were 87 structural alerts identified from the fragments of KRFP fingerprint. A compound would be classified as nephrotoxic if it contains one or more such SAs. These structural alerts showed a good ability to distinguish nephrotoxic drugs in the entire data set. They have been integrated as part of our web server SApredictor, which is freely available at www.sapredictor.cn. The *in silico* models and the structural alerts could be useful tools for estimation of nephrotoxicity in drug discovery.

## Data Availability

The original contributions presented in the study are included in the article/Supplementary Materials, and further inquiries can be directed to the corresponding author.

## References

[B1] Ancuceanu R. Dinu M. Neaga I. Laszlo F. G. Boda D. (2019). Development of QSAR Machine Learning-Based Models to Forecast the Effect of Substances on Malignant Melanoma Cells. Oncol. Lett. 17 (5), 4188–4196. 10.3892/ol.2019.10068 31007759 PMC6466999

[B2] Błaszczak-Świątkiewicz K. Olszewska P. Mikiciuk-Olasik E. (2014). Biological Approach of Anticancer Activity of New Benzimidazole Derivatives. Pharmacol. Rep. 66 (1), 100–106. 10.1016/j.pharep.2014.01.001 24905314

[B3] Breiman L. (2001). Random Forests. Machine Learn. 45 (1), 5–32. 10.1023/a:1010933404324

[B4] Camara-Lemarroy C. R. Rodríguez-Gutiérrez R. Monreal-Robles R. González-González J. G. (2015). Acute Toluene Intoxication-Clinical Presentation, Management and Prognosis: a Prospective Observational Study. BMC Emerg. Med. 15 (1), 19. 10.1186/s12873-015-0039-0 26282250 PMC4539858

[B5] Capela F. Nouchi V. Van Deursen R. Tetko I. V. Godin G. (2019). Multitask Learning on Graph Neural Networks Applied to Molecular Property Predictions. Available at: https://ui.adsabs.harvard.edu/abs/2019arXiv191013124C (Accessed October 01, 2019).

[B6] Chang C.-C. Lin C.-J. (2011). LIBSVM: A Library for Support Vector Machines. ACM Trans. Intell. Syst. Technol. 2, 1–27. 10.1145/1961189.1961199

[B7] Chen T. Guestrin C. (2016). "XGBoost: A Scalable Tree Boosting System", in: Proceedings of the 22nd ACM SIGKDD International Conference on Knowledge Discovery and Data Mining. San Francisco, California, USA: Association for Computing Machinery.

[B8] Claesson A. Minidis A. (2018). Systematic Approach to Organizing Structural Alerts for Reactive Metabolite Formation From Potential Drugs. Chem. Res. Toxicol. 31 (6), 389–411. 10.1021/acs.chemrestox.8b00046 29746101

[B9] Cui X. Liu J. Zhang J. Wu Q. Li X. (2019). In Silico prediction of Drug-Induced Rhabdomyolysis With Machine-Learning Models and Structural Alerts. J. Appl. Toxicol. 39 (8), 1224–1232. 10.1002/jat.3808 31006880

[B10] Cui X. Yang R. Li S. Liu J. Wu Q. Li X. (2021). Modeling and Insights Into Molecular Basis of Low Molecular Weight Respiratory Sensitizers. Mol. Divers. 25 (2), 847–859. 10.1007/s11030-020-10069-3 32166484

[B11] Gai Z. Gui T. Kullak-Ublick G. A. Li Y. Visentin M. (2020). The Role of Mitochondria in Drug-Induced Kidney Injury. Front. Physiol. 11, 1079. 10.3389/fphys.2020.01079 33013462 PMC7500167

[B12] Hall M. Frank E. Holmes G. Pfahringer B. Reutemann P. Witten I. H. (2009). The WEKA Data Mining Software. SIGKDD Explor. Newsl. 11 (1), 10–18. 10.1145/1656274.1656278

[B13] Hörl W. H. (2010). Nonsteroidal Anti-Inflammatory Drugs and the Kidney. Pharmaceuticals. 3, 2291. 10.3390/ph3072291 27713354 PMC4036662

[B14] Hoste E. A. Bagshaw S. M. Bellomo R. Cely C. M. Colman R. Cruz D. N. (2015). Epidemiology of Acute Kidney Injury in Critically Ill Patients: the Multinational AKI-EPI Study. Intensive Care Med. 41 (8), 1411–1423. 10.1007/s00134-015-3934-7 26162677

[B15] Hu Z. Zhang H. Yang S. K. Wu X. He D. Cao K. (2019). Emerging Role of Ferroptosis in Acute Kidney Injury. Oxid Med. Cell Longev. 2019, 8010614. 10.1155/2019/8010614 31781351 PMC6875218

[B16] Hua Y. Shi Y. Cui X. Li X. (2021). In Silico prediction of Chemical-Induced Hematotoxicity with Machine Learning and Deep Learning Methods. Mol. Divers. 25 (3), 1585–1596. 10.1007/s11030-021-10255-x 34196933

[B17] Huang X. Tang F. Hua Y. Li X. (2021). In Silico Prediction of Drug-Induced Ototoxicity Using Machine Learning and Deep Learning Methods. Chem. Biol. Drug Des. 98 (2), 248–257. 10.1111/cbdd.13894 34013639

[B18] Jolliffe I. T. Cadima J. (2016). Principal Component Analysis: a Review and Recent Developments. Philos. Trans. A. Math. Phys. Eng. Sci. 374 (2065), 20150202. 10.1098/rsta.2015.0202 26953178 PMC4792409

[B19] Karpov P. Godin G. Tetko I. V. (2020). Transformer-CNN: Swiss Knife for QSAR Modeling and Interpretation. J. Cheminform. 12 (1), 17. 10.1186/s13321-020-00423-w 33431004 PMC7079452

[B20] Kim S. Thiessen P. A. Bolton E. E. Chen J. Fu G. Gindulyte A. (2016). PubChem Substance and Compound Databases. Nucleic Acids Res. 44 (D1), D1202–D1213. 10.1093/nar/gkv951 26400175 PMC4702940

[B21] Kuhn M. Letunic I. Jensen L. J. Bork P. (2016). The SIDER Database of Drugs and Side Effects. Nucleic Acids Res. 44 (D1), D1075–D1079. 10.1093/nar/gkv1075 26481350 PMC4702794

[B22] Kwiatkowska E. Domański L. Dziedziejko V. Kajdy A. Stefańska K. Kwiatkowski S. (2021). The Mechanism of Drug Nephrotoxicity and the Methods for Preventing Kidney Damage. Int. J. Mol. Sci. 22 (11), 6109. 10.3390/ijms22116109 34204029 PMC8201165

[B23] Lei T. Sun H. Kang Y. Zhu F. Liu H. Zhou W. (2017). ADMET Evaluation in Drug Discovery. 18. Reliable Prediction of Chemical-Induced Urinary Tract Toxicity by Boosting Machine Learning Approaches. Mol. Pharm. 14 (11), 3935–3953. 10.1021/acs.molpharmaceut.7b00631 29037046

[B24] Li J. Cao F. Yin H. L. Huang Z. J. Lin Z. T. Mao N. (2020). Ferroptosis: Past, Present and Future. Cell Death Dis. 11 (2), 88. 10.1038/s41419-020-2298-2 32015325 PMC6997353

[B25] Mehta R. L. Pascual M. T. Soroko S. Savage B. R. Himmelfarb J. Ikizler T. A. (2004). Spectrum of Acute Renal Failure in the Intensive Care Unit: The PICARD Experience. Kidney Int. 66 (4), 1613–1621. 10.1111/j.1523-1755.2004.00927.x 15458458

[B26] Murray Stewart T. Dunston T. T. Woster P. M. Casero R. A. Jr (2018). Polyamine Catabolism and Oxidative Damage. J. Biol. Chem. 293 (48), 18736–18745. 10.1074/jbc.TM118.003337 30333229 PMC6290137

[B27] Nolin T. D. Himmelfarb J. (2010). Mechanisms of Drug-Induced Nephrotoxicity. Adverse Drug React. 196, 111–130. 10.1007/978-3-642-00663-0_5 20020261

[B28] Oprisiu I. Novotarskyi S. Tetko I. V. (2013). Modeling of Non-Additive Mixture Properties Using the Online CHEmical Database and Modeling Environment (OCHEM). J. Cheminform. 5 (1), 4. 10.1186/1758-2946-5-4 23321019 PMC3568005

[B29] Ou Y. Wang S. J. Li D. Chu B. Gu W. (2016). Activation of SAT1 Engages Polyamine Metabolism With P53-Mediated Ferroptotic Responses. Proc. Natl. Acad. Sci. U S A. 113 (44), E6806. 10.1073/pnas.1607152113 27698118 PMC5098629

[B30] Paller M. S. Hoidal J. R. Ferris T. F. (1984). Oxygen Free Radicals in Ischemic Acute Renal Failure in the Rat. J. Clin. Invest. 74 (4), 1156–1164. 10.1172/JCI111524 6434591 PMC425281

[B31] Pan Y. (2019). The Dark Side of Fluorine. ACS Med. Chem. Lett. 10 (7), 1016–1019. 10.1021/acsmedchemlett.9b00235 31312400 PMC6627733

[B32] Pawar G. Madden J. C. Ebbrell D. Firman J. W. Cronin M. T. D. (2019). Silico Toxicology Data Resources to Support Read-Across and (Q)SAR. Front. Pharmacol. 10, 561. 10.3389/fphar.2019.00561 31244651 PMC6580867

[B33] Pegg A. E. (2013). Toxicity of Polyamines and Their Metabolic Products. Chem. Res. Toxicol. 26 (12), 1782–1800. 10.1021/tx400316s 24224555

[B34] Quadri J. A. Alam M. M. Sarwar S. Singh S. Shariff A. Das T. K. (2016). Fluoride Induced Nephrotoxicity: Apoptosis, Ultrastructural Changes and Renal Tubular Injury in Experimental Animals. Int. J. Ayurveda Pharma Res. 4, 91–95.

[B35] Raza Z. Naureen Z. (2020). Melatonin Ameliorates the Drug Induced Nephrotoxicity: Molecular Insights. Nefrologia. 40 (1), 12–25. 10.1016/j.nefro.2019.06.009 31735377

[B36] Ren L.-w. Li W. Zheng X.-j. Liu J.-y. Yang Y.-h. Li S. (2021). Benzimidazoles Induce Concurrent Apoptosis and Pyroptosis of Human Glioblastoma Cells via Arresting Cell Cycle. Acta Pharmacologica Sinica. 10.1038/s41401-021-00752-y PMC8724275 34433903

[B37] Ringnér M. (2008). What Is Principal Component Analysis? Nat. Biotechnol. 26 (3), 303–304. 10.1038/nbt0308-303 18327243

[B38] Sales G. T. M. Foresto R. D. (2020). Drug-Induced Nephrotoxicity. Rev. Assoc. Med. Bras. 66 (Suppl. l), s82–s90. 10.1590/1806-9282.66.s1.82 31939540

[B39] Sekine T. Endou H. (2009). Children's Toxicology from Bench to Bed--DrugIinduced Renal Injury (3): Drug Transporters and Toxic Nephropathy in Childhood. J. Toxicol. Sci. 34, SP259–65. 10.2131/jts.34.SP259 19571478

[B40] Shang C. Liu Q. Chen K.-S. Sun J. Lu J. Yi J. (2018). Edge Attention-Based Multi-Relational Graph Convolutional Networks. Available. Available at: https://ui.adsabs.harvard.edu/abs/2018arXiv180204944S (Accessed February 01, 2018).

[B41] Spanou Z. Keller M. Britschgi M. Yawalkar N. Fehr T. Neuweiler J. (2006). Involvement of Drug-Specific T Cells in Acute Drug-Induced Interstitial Nephritis. J. Am. Soc. Nephrol. 17 (10), 2919–2927. 10.1681/ASN.2006050418 16943303

[B42] Sun Y. Shi S. Li Y. Wang Q. (2019). Development of Quantitative Structure-Activity Relationship Models to Predict Potential Nephrotoxic Ingredients in Traditional Chinese Medicines. Food Chem. Toxicol. 128, 163–170. 10.1016/j.fct.2019.03.056 30954639

[B43] Sushko I. Novotarskyi S. Körner R. Pandey A. K. Rupp M. Teetz W. (2011). Online Chemical Modeling Environment (OCHEM): Web Platform for Data Storage, Model Development and Publishing of Chemical Information. J. Comput. Aided Mol. Des. 25 (6), 533–554. 10.1007/s10822-011-9440-2 21660515 PMC3131510

[B44] Ta G. H. Weng C.-F. Leong M. K. (2021). In Silico Prediction of Skin Sensitization: Quo Vadis? Front. Pharmacol. 12, 655771. 10.3389/fphar.2021.655771 34017255 PMC8129647

[B45] Tapia Garcı’a J. M. del Moral M. J. Martínez M. A. Herrera-Viedma E. (2012). A Consensus Model for Group Decision Making Problems with Linguistic Interval Fuzzy Preference Relations. Expert Syst. Appl. 39 (11), 10022–10030. 10.1016/j.eswa.2012.02.008

[B46] Tetko I. V. (2009). “Associative Neural Network,” in Artificial Neural Networks: Methods and Applications. Editors Livingstone D. J., and (Totowa, NJ: Humana Press), 180–197.

[B47] Tetko I. V. Novotarskyi S. Sushko I. Ivanov V. Petrenko A. E. Dieden R. (2013). Development of Dimethyl Sulfoxide Solubility Models Using 163,000 Molecules: Using a Domain Applicability Metric to Select More Reliable Predictions. J. Chem. Inf. Model. 53 (8), 1990–2000. 10.1021/ci400213d 23855787 PMC3760295

[B48] Tetko I. V. Karpov P. Bruno E. Kimber T. B. Godin G. (2019). Augmentation Is What You Need!. Lecture Notes Computer Sci. 11731, 831. 10.1007/978-3-030-30493-5_79

[B49] Thormann M. Vidal D. Almstetter M. Pons M. (2007). Nomen Est Omen: Quantitative Prediction of Molecular Properties Directly from IUPAC Names. Toainfoj. 1, 28–32. 10.2174/1874136300701010028

[B50] Uchino S. Kellum J. A. Bellomo R. Doig G. S. Morimatsu H. Morgera S. (2005). Acute Renal Failure in Critically Ill Patients: a Multinational, Multicenter Study. JAMA. 294 (7), 813–818. 10.1001/jama.294.7.813 16106006

[B51] Wedlake A. J. Allen T. E. H. Goodman J. M. Gutsell S. Kukic P. Russell P. J. (2020). Confidence in Inactive and Active Predictions From Structural Alerts. Chem. Res. Toxicol. 33 (12), 3010–3022. 10.1021/acs.chemrestox.0c00332 33295767

[B52] Wu Z. Ramsundar B. Feinberg E. N. Gomes J. Geniesse C. Pappu A. S. (2017). MoleculeNet: A Benchmark for Molecular Machine Learning. Available. Available at: https://ui.adsabs.harvard.edu/abs/2017arXiv170300564W (Accessed March 01, 2017). 10.1039/c7sc02664a PMC5868307 29629118

[B53] Yang H. Sun L. Li W. Liu G. Tang Y. (2018). Identification of Nontoxic Substructures: A New Strategy to Avoid Potential Toxicity Risk. Toxicol. Sci. 165 (2), 396–407. 10.1093/toxsci/kfy146 29893961

[B54] Yap C. W. (2011). PaDEL-descriptor: An Open Source Software to Calculate Molecular Descriptors and Fingerprints. J. Comput. Chem. 32 (7), 1466–1474. 10.1002/jcc.21707 21425294

[B55] Zhang H. Ren J. X. Ma J. X. Ding L. (2019). Development of an In Silico Prediction Model for Chemical-Induced Urinary Tract Toxicity by Using Naïve Bayes Classifier. Mol. Divers. 23 (2), 381–392. 10.1007/s11030-018-9882-8 30294757

