# Supplementary material for: In Silico Prediction and Insights Into the Structural Basis of Drug Induced Nephrotoxicity
Source: Front Pharmacol. 2022 Jan 5;12:793332. doi: 10.3389/fphar.2021.793332 (PMC8785686; doi:10.3389/fphar.2021.793332)
Supplement: Supplementary file 1 [file DataSheet1.DOCX]

Table S1 Drugs for model building and validation

| **Name** | **Label** | **Dataset** | **SMILES** |
| --- | --- | --- | --- |
| Risperidone | nephrotoxic | training | CC1=C(CCN2CCC(CC2)C2=NOC3=CC(F)=CC=C23)C(=O)N2CCCCC2=N1 |
| Pantoprazole | nephrotoxic | training | COC1=CC=NC(CS(=O)C2=NC3=CC=C(OC(F)F)C=C3N2)=C1OC |
| Miglustat | non-nephrotoxic | training | CCCCN1C[C@H](O)[C@@H](O)[C@H](O)[C@H]1CO |
| Ethacrynic acid | non-nephrotoxic | training | CCC(=C)C(=O)C1=CC=C(OCC(O)=O)C(Cl)=C1Cl |
| Rimonabant hydrochloride | non-nephrotoxic | training | CC1=C(N(N=C1C(=O)NN1CCCCC1)C1=CC=C(Cl)C=C1Cl)C1=CC=C(Cl)C=C1 |
| Valsartan | nephrotoxic | training | CCCCC(=O)N(CC1=CC=C(C=C1)C1=C(C=CC=C1)C1=NNN=N1)[C@@H](C(C)C)C(O)=O |
| Captopril | nephrotoxic | training | C[C@H](CS)C(=O)N1CCC[C@H]1C(O)=O |
| Triaconazole | non-nephrotoxic | training | CC(C)N1CCN(CC1)C1=CC=C(OCC2COC(CN3C=NC=N3)(O2)C2=CC=C(Cl)C=C2Cl)C=C1 |
| Isotretinoin | nephrotoxic | training | C\C(\C=C\C1=C(C)CCCC1(C)C)=C/C=C/C(/C)=C\C(O)=O |
| entecavir | nephrotoxic | training | OC[C@@H]1[C@@H](O)C[C@H](N2C=NC3=C2N=CNC3=O)C1=C |
| Procaine | non-nephrotoxic | training | CCN(CC)CCOC(=O)C1=CC=C(N)C=C1 |
| Hexachlorophene | non-nephrotoxic | training | OC1=C(Cl)C=C(Cl)C(Cl)=C1CC1=C(Cl)C(Cl)=CC(Cl)=C1O |
| Ezetimibe | nephrotoxic | training | O[C@@H](CC[C@@H]1[C@H](N(C1=O)C1=CC=C(F)C=C1)C1=CC=C(O)C=C1)C1=CC=C(F)C=C1 |
| Lactulose | non-nephrotoxic | training | OCC1OC(O)(CO)C(O)C1OC1OC(CO)C(O)C(O)C1O |
| Ibutilide (fumarate) | nephrotoxic | training | CCCCCCCN(CC)CCCC(O)C1=CC=C(NS(C)(=O)=O)C=C1 |
| Gadodiamide | nephrotoxic | training | CNC(=O)CN(CCN(CCN(CC([O-])=O)CC(=O)NC)CC([O-])=O)CC([O-])=O |
| Betaine | non-nephrotoxic | training | C[N+](C)(C)CC([O-])=O |
| Sevoflurane | nephrotoxic | training | FCOC(C(F)(F)F)C(F)(F)F |
| Regroton | non-nephrotoxic | training | COC1C(CC2CN3CCC4=C(NC5=CC(OC)=CC=C45)C3CC2C1C(=O)OC)OC(=O)C1=CC(OC)=C(OC)C(OC)=C1 |
| Cefpodoxime | nephrotoxic | training | COCC1=C(N2[C@H](SC1)[C@H](NC(=O)C(=N/OC)\C1=CSC(N)=N1)C2=O)C(O)=O |
| Elocom | non-nephrotoxic | training | C[C@@H]1C[C@H]2C3CCC4=CC(=O)C=C[C@]4(C)[C@@]3(Cl)[C@@H](O)C[C@]2(C)[C@@]1(O)C(=O)CCl |
| Omeprazole | nephrotoxic | training | COC1=CC=C2N=C(NC2=C1)S(=O)CC1=C(C)C(OC)=C(C)C=N1 |
| Prostacycline | non-nephrotoxic | training | CCCCCC(O)C=CC1C(O)CC2OC(CC12)=CCCCC(O)=O |
| Streptozocin | nephrotoxic | training | CN(N=O)C(=O)N[C@H]1[C@@H](O)O[C@H](CO)[C@@H](O)[C@@H]1O |
| Quetiapine | nephrotoxic | training | OCCOCCN1CCN(CC1)C1=NC2=C(SC3=C1C=CC=C3)C=CC=C2 |
| Donepezil | nephrotoxic | training | COC1=CC2=C(C=C1OC)C(=O)C(CC1CCN(CC3=CC=CC=C3)CC1)C2 |
| Lovastatin | nephrotoxic | training | CC[C@H](C)C(=O)O[C@H]1C[C@@H](C)C=C2C=C[C@H](C)[C@H](CC[C@@H]3C[C@@H](O)CC(=O)O3)[C@@H]12 |
| Aspirin | non-nephrotoxic | training | CC(=O)OC1=C(C=CC=C1)C(O)=O |
| Letrozole | nephrotoxic | training | N#CC1=CC=C(C=C1)C(N1C=NC=N1)C1=CC=C(C=C1)C#N |
| Tenofovir disoproxil | nephrotoxic | training | CC(C)OC(=O)OCOP(=O)(CO[C@H](C)CN1C=NC2=C(N)N=CN=C12)OCOC(=O)OC(C)C |
| Trametinib | nephrotoxic | training | CN1C(=O)C(C)=C2N(C(=O)N(C3CC3)C(=O)C2=C1NC1=CC=C(I)C=C1F)C1=CC(NC(C)=O)=CC=C1 |
| Methimazole | non-nephrotoxic | training | CN1C=CNC1=S |
| Regurin | nephrotoxic | training | OC(C(=O)OC1CC2CCC(C1)[N+]21CCCC1)(C1=CC=CC=C1)C1=CC=CC=C1 |
| Sunitinib | nephrotoxic | training | CCN(CC)CCNC(=O)C1=C(C)NC(\C=C2/C(=O)NC3=CC=C(F)C=C23)=C1C |
| Perindopril erbumine | nephrotoxic | training | CCC[C@H](N[C@@H](C)C(=O)N1[C@H]2CCCC[C@H]2C[C@H]1C(O)=O)C(=O)OCC |
| Oxytocin | non-nephrotoxic | training | CCC(C)C1NC(=O)C(CC2=CC=C(O)C=C2)NC(=O)C(N)CSSCC(NC(=O)C(CC(N)=O)NC(=O)C(CCC(N)=O)NC1=O)C(=O)N1CCCC1C(=O)NC(CC(C)C)C(=O)NCC(N)=O |
| Prasugrel | non-nephrotoxic | training | CC(=O)OC1=CC2=C(CCN(C2)C(C(=O)C2CC2)C2=C(F)C=CC=C2)S1 |
| Perindoprilat | nephrotoxic | training | CCC[C@H](N[C@@H](C)C(=O)N1[C@H]2CCCC[C@H]2C[C@H]1C(O)=O)C(O)=O |
| Nabilone | non-nephrotoxic | training | CCCCCCC(C)(C)C1=CC2=C(C3CC(=O)CCC3C(C)(C)O2)C(O)=C1 |
| (-)-Prostaglandin E2 | non-nephrotoxic | training | CCCCCC(O)C=CC1C(O)CC(=O)C1CC=CCCCC(O)=O |
| Eszopiclone | nephrotoxic | training | CN1CCN(CC1)C(=O)O[C@@H]1N(C(=O)C2=C1N=CC=N2)C1=CC=C(Cl)C=N1 |
| Lamisil | nephrotoxic | training | C[NH+](C\C=C\C#CC(C)(C)C)CC1=C2C=CC=CC2=CC=C1 |
| Mirtazapine | nephrotoxic | training | CN1CCN2C(C1)C1=C(CC3=C2N=CC=C3)C=CC=C1 |
| Caffeine | nephrotoxic | training | CN1C=NC2=C1C(=O)N(C)C(=O)N2C |
| anagrelide | nephrotoxic | training | ClC1=C(Cl)C2=C(C=C1)N=C1NC(=O)CN1C2 |
| 2-[(E)-[5-Methoxy-1-[4-(trifluoromethyl)phenyl]pentylidene]amino]oxyethylazanium | nephrotoxic | training | COCCCC\C(=N/OCC[NH3+])C1=CC=C(C=C1)C(F)(F)F |
| Monobenzone | non-nephrotoxic | training | OC1=CC=C(OCC2=CC=CC=C2)C=C1 |
| Troglitazone | non-nephrotoxic | training | CC1=C(O)C(C)=C2CCC(C)(COC3=CC=C(CC4SC(=O)NC4=O)C=C3)OC2=C1C |
| Penbuterol | non-nephrotoxic | training | CC(C)(C)NCC(O)COC1=C(C=CC=C1)C1CCCC1 |
| Gatifloxacin | nephrotoxic | training | COC1=C(N2CCNC(C)C2)C(F)=CC2=C1N(C=C(C(O)=O)C2=O)C1CC1 |
| Pindolol | non-nephrotoxic | training | CC(C)NCC(O)COC1=C2C=CNC2=CC=C1 |
| Hydrocortisone butyrate | non-nephrotoxic | training | CCCC(=O)OC1(CCC2C3CCC4=CC(=O)CCC4(C)C3C(O)CC12C)C(=O)CO |
| Amlodipine | non-nephrotoxic | training | CCOC(=O)C1=C(COCCN)NC(C)=C(C1C1=C(Cl)C=CC=C1)C(=O)OC |
| (1S,2R,18S,19R,22S,25R,28R,40S)-22-(2-Amino-2-oxoethyl)-5,15-dichloro-48-[(2S,3R,4S,5S,6R)-3-[(2S,4S,5S,6S)-4-[2-(decylamino)ethylamino]-5-hydroxy-4,6-dimethyloxan-2-yl]oxy-4,5-dihydroxy-6-(hydroxymethyl)oxan-2-yl]oxy-2,18,32,35,37-pentahydroxy-19-[[(2R)-4-methyl-2-(methylamino)pentanoyl]amino]-20,23,26,42,44-pentaoxo-36-[(phosphonomethylamino)methyl]-7,13-dioxa-21,24,27,41,43-pentazaoctacyclo[26.14.2.23,6.214,17.18,12.129,33.010,25.034,39]pentaconta-3,5,8(48),9,11,14,16,29(45),30,32,34,36,38,46,49-pentadecaene-40-carboxylic acid | nephrotoxic | training | CCCCCCCCCCNCCN[C@@]1(C)C[C@H](O[C@@H]2[C@@H](O)[C@H](O)[C@@H](CO)O[C@H]2OC2=C3OC4=CC=C(C=C4Cl)[C@H](O)[C@@H](NC(=O)[C@@H](CC(C)C)NC)C(=O)N[C@@H](CC(N)=O)C(=O)N[C@@H]4C(C=C2OC2=C(Cl)C=C(C=C2)[C@@H](O)[C@@H]2NC(=O)[C@H](NC4=O)C4=CC=C(O)C(=C4)C4=C(C=C(O)C(CNCP(O)(O)=O)=C4O)[C@H](NC2=O)C(O)=O)=C3)O[C@@H](C)[C@H]1O |
| Acarbose | non-nephrotoxic | training | C[C@H]1O[C@H](O[C@@H]2[C@@H](CO)O[C@H](O[C@@H]3[C@@H](CO)OC(O)[C@H](O)[C@H]3O)[C@H](O)[C@H]2O)[C@H](O)[C@@H](O)[C@@H]1N[C@H]1C=C(CO)[C@@H](O)[C@H](O)[C@H]1O |
| Tolazamide | non-nephrotoxic | training | CC1=CC=C(C=C1)S(=O)(=O)NC(=O)NN1CCCCCC1 |
| Lasofoxifene | nephrotoxic | training | OC1=CC2=C(C=C1)[C@H]([C@H](CC2)C1=CC=CC=C1)C1=CC=C(OCCN2CCCC2)C=C1 |
| Losartan | nephrotoxic | training | CCCCC1=NC(Cl)=C(CO)N1CC1=CC=C(C=C1)C1=C(C=CC=C1)C1=NNN=N1 |
| 9-cis Acitretin | non-nephrotoxic | training | COC1=CC(C)=C(C=CC(C)=CC=CC(C)=CC(O)=O)C(C)=C1C |
| Salsalate | non-nephrotoxic | training | OC(=O)C1=C(OC(=O)C2=C(O)C=CC=C2)C=CC=C1 |
| Cobicistat | nephrotoxic | training | CC(C)C1=NC(CN(C)C(=O)N[C@@H](CCN2CCOCC2)C(=O)N[C@H](CC[C@H](CC2=CC=CC=C2)NC(=O)OCC2=CN=CS2)CC2=CC=CC=C2)=CS1 |
| Micafungin | nephrotoxic | training | CCCCCOC1=CC=C(C=C1)C1=CC(=NO1)C1=CC=C(C=C1)C(=O)N[C@H]1C[C@@H](O)[C@@H](O)NC(=O)[C@@H]2[C@@H](O)[C@@H](C)CN2C(=O)[C@@H](NC(=O)[C@@H](NC(=O)[C@@H]2C[C@@H](O)CN2C(=O)[C@@H](NC1=O)[C@@H](C)O)[C@H](O)[C@@H](O)C1=CC=C(O)C(OS(O)(=O)=O)=C1)[C@H](O)CC(N)=O |
| Indapamide | nephrotoxic | training | CC1CC2=C(C=CC=C2)N1NC(=O)C1=CC=C(Cl)C(=C1)S(N)(=O)=O |
| Iopromide | nephrotoxic | training | COCC(=O)NC1=C(I)C(C(=O)N(C)CC(O)CO)=C(I)C(C(=O)NCC(O)CO)=C1I |
| Dekamycin II | non-nephrotoxic | training | NCC1OC(OC2C(CO)OC(OC3C(O)C(N)CC(N)C3OC3OC(CN)C(O)C(O)C3N)C2O)C(N)C(O)C1O |
| Candesartan cilexetil | nephrotoxic | training | CCOC1=NC2=CC=CC(C(=O)OC(C)OC(=O)OC3CCCCC3)=C2N1CC1=CC=C(C=C1)C1=C(C=CC=C1)C1=NNN=N1 |
| Etravirine | nephrotoxic | training | CC1=CC(=CC(C)=C1OC1=C(Br)C(N)=NC(NC2=CC=C(C=C2)C#N)=N1)C#N |
| Bonzol | non-nephrotoxic | training | CC12CCC3C(CCC4=CC5=C(CC34C)C=NO5)C1CCC2(O)C#C |
| Bupropion | nephrotoxic | training | CC(NC(C)(C)C)C(=O)C1=CC=CC(Cl)=C1 |
| Milrinone | non-nephrotoxic | training | CC1=C(C=C(C#N)C(=O)N1)C1=CC=NC=C1 |
| Itraconazole | nephrotoxic | training | CCC(C)N1N=CN(C1=O)C1=CC=C(C=C1)N1CCN(CC1)C1=CC=C(OC[C@H]2CO[C@@](CN3C=NC=N3)(O2)C2=CC=C(Cl)C=C2Cl)C=C1 |
| Natamycin | non-nephrotoxic | training | C[C@H]1OC(O[C@@H]2C[C@@H]3O[C@@](O)(C[C@H](O)[C@H]3C(O)=O)C[C@@H](O)C[C@H]3O[C@@H]3C=CC(=O)O[C@H](C)CC=CC=CC=CC=C2)[C@@H](O)[C@@H](N)[C@@H]1O |
| Cubicin | nephrotoxic | training | CCCCCCCCCC(=O)N[C@@H](CC1=CNC2=CC=CC=C12)C(=O)N[C@@H](CC(N)=O)C(=O)N[C@@H](CC(O)=O)C(=O)NC1C(C)OC(=O)[C@H](CC(=O)C2=C(N)C=CC=C2)NC(=O)[C@@H](NC(=O)[C@@H](CO)NC(=O)CNC(=O)[C@H](CC(O)=O)NC(=O)[C@@H](C)NC(=O)[C@H](CC(O)=O)NC(=O)[C@H](CCCN)NC(=O)CNC1=O)C(C)CC(O)=O |
| Candesartan | nephrotoxic | training | CCOC1=NC2=CC=CC(C(O)=O)=C2N1CC1=CC=C(C=C1)C1=C(C=CC=C1)C1=NNN=N1 |
| Chlorzoxazone | non-nephrotoxic | training | ClC1=CC=C2OC(=O)NC2=C1 |
| Desflurane | nephrotoxic | training | FC(F)OC(F)C(F)(F)F |
| Lubiprostone | non-nephrotoxic | training | CCCCC(F)(F)[C@@]1(O)CC[C@H]2[C@@H](CC(=O)[C@@H]2CCCCCCC(O)=O)O1 |
| Metolazone | non-nephrotoxic | training | CC1NC2=C(C=C(C(Cl)=C2)S(N)(=O)=O)C(=O)N1C1=C(C)C=CC=C1 |
| Clindamycin | non-nephrotoxic | training | CCCC1CC(N(C)C1)C(=O)NC(C(C)Cl)C1OC(SC)C(O)C(O)C1O |
| Mecamylamine | non-nephrotoxic | training | CNC1(C)C2CCC(C2)C1(C)C |
| Cefditoren pivoxil | nephrotoxic | training | CON=C(C(=O)N[C@H]1[C@H]2SCC(C=CC3=C(C)N=CS3)=C(N2C1=O)C(=O)OCOC(=O)C(C)(C)C)C1=CSC(N)=N1 |
| Silvadene | non-nephrotoxic | training | NC1=CC=C(C=C1)S(=O)(=[OH+])[NH2+]C1=NC=CC=N1 |
| (1R,9S,12S,13R,14S,17R,18E,21S,23S,24R,25S,27R)-1,14-Dihydroxy-12-[(E)-1-[(3R,4R)-4-hydroxy-3-methoxycyclohexyl]prop-1-en-2-yl]-23,25-dimethoxy-13,19,21,27-tetramethyl-17-prop-2-enyl-11,28-dioxa-4-azatricyclo[22.3.1.04,9]octacos-18-ene-2,3,10,16-tetrone;hydrate | nephrotoxic | training | CO[C@@H]1CC(CC[C@H]1O)\C=C(/C)[C@H]1OC(=O)[C@@H]2CCCCN2C(=O)C(=O)[C@]2(O)O[C@@H]([C@H](C[C@H]2C)OC)[C@H](C[C@@H](C)C\C(C)=C/[C@@H](CC=C)C(=O)C[C@H](O)[C@H]1C)OC |
| Finasteride | non-nephrotoxic | training | CC(C)(C)NC(=O)C1CCC2C3CCC4NC(=O)C=CC4(C)C3CCC12C |
| Adefovir | nephrotoxic | training | NC1=C2N=CN(CCOCP(O)(O)=O)C2=NC=N1 |
| Mycophenolic acid | nephrotoxic | training | COC1=C(C)C2=C(C(=O)OC2)C(O)=C1C\C=C(/C)CCC(O)=O |
| Hydralazine | nephrotoxic | training | NNC1=NN=CC2=CC=CC=C12 |
| Prucalopride | non-nephrotoxic | training | COCCCN1CCC(CC1)NC(=O)C1=C2OCCC2=C(N)C(Cl)=C1 |
| Phenylpropanolamine | non-nephrotoxic | training | CC(N)C(O)C1=CC=CC=C1 |
| Urofollitropin | non-nephrotoxic | training | CCC(C)C1NC(=O)C(CC2=CC=C(O)C=C2)NC(=O)C(N)CSSCC(NC(=O)C(CC(N)=O)NC(=O)C(NC1=O)C(C)O)C(=O)N1CCCC1C(=O)NC(CC(C)C)C(=O)NCC(N)=O |
| Escitalopram | nephrotoxic | training | CN(C)CCC[C@]1(OCC2=C1C=CC(=C2)C#N)C1=CC=C(F)C=C1 |
| Rosuvastatin | nephrotoxic | training | CC(C)C1=C(\C=C\[C@@H](O)C[C@@H](O)CC(O)=O)C(=NC(=N1)N(C)S(C)(=O)=O)C1=CC=C(F)C=C1 |
| 6alpha-Methylcompactin | non-nephrotoxic | training | CCC(C)C(=O)OC1CC(C)C=C2C=CC(C)C(CCC3CC(O)CC(=O)O3)C12 |
| Heparin | non-nephrotoxic | training | CC(=O)NC1C(O)OC(COS(O)(=O)=O)C(OC2OC(C(OC3OC(CO)C(OC4OC(C(O)C(O)C4OS(O)(=O)=O)C(O)=O)C(OS(O)(=O)=O)C3NS(O)(=O)=O)C(O)C2OS(O)(=O)=O)C(O)=O)C1O |
| Glimepiride | non-nephrotoxic | training | CCC1=C(C)CN(C(=O)NCCC2=CC=C(C=C2)S(=O)(=O)NC(=O)NC2CCC(C)CC2)C1=O |
| Articaine | non-nephrotoxic | training | CCCNC(C)C(=O)NC1=C(SC=C1C)C(=O)OC |
| Argatroban monohydrate | nephrotoxic | training | C[C@@H]1CCN([C@H](C1)C(O)=O)C(=O)[C@H](CCCN=C(N)N)NS(=O)(=O)C1=C2NCC(C)CC2=CC=C1 |
| Meloxicam sodium | nephrotoxic | training | CN1C(C(=O)NC2=NC=C(C)S2)=C([O-])C2=C(C=CC=C2)S1(=O)=O |
| Colesevelam hydrochloride | non-nephrotoxic | training | CCCCCCCCCCNCC=C |
| Vasopressin tannate | non-nephrotoxic | training | NC1CSSCC(NC(=O)C(CC(N)=O)NC(=O)C(CCC(N)=O)NC(=O)C(CC2=CC=CC=C2)NC(=O)C(CC2=CC=C(O)C=C2)NC1=O)C(=O)N1CCCC1C(=O)NC(CCCN=C(N)N)C(=O)NCC(N)=O |
| Telaprevir | nephrotoxic | training | CCC[C@H](NC(=O)[C@@H]1[C@H]2CCC[C@H]2CN1C(=O)[C@@H](NC(=O)[C@@H](NC(=O)C1=CN=CC=N1)C1CCCCC1)C(C)(C)C)C(=O)C(=O)NC1CC1 |
| Lamivudine | nephrotoxic | training | NC1=NC(=O)N(C=C1)[C@@H]1CS[C@H](CO)O1 |
| Phentolamine | non-nephrotoxic | training | CC1=CC=C(C=C1)N(CC1=NCCN1)C1=CC=CC(O)=C1 |
| Amantadine | nephrotoxic | training | NC12CC3CC(CC(C3)C1)C2 |
| Alfuzosin | nephrotoxic | training | COC1=CC2=NC(=NC(N)=C2C=C1OC)N(C)CCCNC(=O)C1CCCO1 |
| Pentoxifylline | non-nephrotoxic | training | CN1C=NC2=C1C(=O)N(CCCCC(C)=O)C(=O)N2C |
| Albendazole | nephrotoxic | training | CCCSC1=CC=C2N=C(NC(=O)OC)NC2=C1 |
| Pulmicort | non-nephrotoxic | training | CCCC1OC2CC3C4CCC5=CC(=O)C=CC5(C)C4C(O)CC3(C)C2(O1)C(=O)CO |
| Enflurane | non-nephrotoxic | training | FC(F)OC(F)(F)C(F)Cl |
| Phentermine | non-nephrotoxic | training | CC(C)(N)CC1=CC=CC=C1 |
| Pralatrexate | nephrotoxic | training | NC1=NC2=NC=C(CC(CC#C)C3=CC=C(C=C3)C(=O)N[C@@H](CCC(O)=O)C(O)=O)N=C2C(N)=N1 |
| Halcort | non-nephrotoxic | training | CC1(C)OC2CC3C4CCC5=CC(=O)CCC5(C)C4(F)C(O)CC3(C)C2(O1)C(=O)CCl |
| Nitisinone | non-nephrotoxic | training | [O-][N+](=O)C1=CC(=CC=C1C(=O)C1C(=O)CCCC1=O)C(F)(F)F |
| Cinacalcet hydrochloride | nephrotoxic | training | C[C@@H](NCCCC1=CC=CC(=C1)C(F)(F)F)C1=C2C=CC=CC2=CC=C1 |
| Dihydrocodeine | non-nephrotoxic | training | COC1=CC=C2CC3C4CCC(O)C5OC1=C2C45CCN3C |
| Fludroxycortida | non-nephrotoxic | training | CC1(C)OC2CC3C4CC(F)C5=CC(=O)CCC5(C)C4C(O)CC3(C)C2(O1)C(=O)CO |
| Acebutolol | non-nephrotoxic | training | CCCC(=O)NC1=CC=C(OCC(O)CNC(C)C)C(=C1)C(C)=O |
| Docetaxel trihydrate | nephrotoxic | training | CC(=O)O[C@@]12CO[C@@H]1C[C@H](O)[C@]1(C)[C@@H]2[C@H](OC(=O)C2=CC=CC=C2)[C@]2(O)C[C@H](OC(=O)[C@H](O)[C@@H](NC(=O)OC(C)(C)C)C3=CC=CC=C3)C(C)=C([C@@H](O)C1=O)C2(C)C |
| Acetate | non-nephrotoxic | training | CC([O-])=O |
| Colchicine | nephrotoxic | training | COC1=CC2=C(C(OC)=C1OC)C1=CC=C(OC)C(=O)C=C1[C@H](CC2)NC(C)=O |
| Atovaquone | nephrotoxic | training | OC1=C(C2CCC(CC2)C2=CC=C(Cl)C=C2)C(=O)C(=O)C2=C1C=CC=C2 |
| Polymyxin b | non-nephrotoxic | training | CCC(C)CCCCC(=O)NC(CCN)C(=O)NC(C(C)O)C(=O)NC(CCN)C(=O)NC1CCNC(=O)C(NC(=O)C(CCN)NC(=O)C(CCN)NC(=O)C(CC(C)C)NC(=O)C(CC2=CC=CC=C2)NC(=O)C(CCN)NC1=O)C(C)O |
| [(1S,2R)-2-(Diethylcarbamoyl)-2-phenylcyclopropyl]methylazanium;chloride | nephrotoxic | training | CCN(CC)C(=O)[C@@]1(C[C@@H]1C[NH3+])C1=CC=CC=C1 |
| Cefepime | nephrotoxic | training | CO\N=C(/C(=O)N[C@H]1[C@H]2SCC(C[N+]3(C)CCCC3)=C(N2C1=O)C([O-])=O)C1=CSC(N)=N1 |
| Dabrafenib | nephrotoxic | training | CC(C)(C)C1=NC(=C(S1)C1=CC=NC(N)=N1)C1=CC=CC(NS(=O)(=O)C2=C(F)C=CC=C2F)=C1F |
| [2-[4-[(2-Butylbenzofuran-3-yl)carbonyl]-2,6-diiodophenoxy]ethyl]diethylaminium | nephrotoxic | training | CCCCC1=C(C(=O)C2=CC(I)=C(OCC[NH+](CC)CC)C(I)=C2)C2=CC=CC=C2O1 |
| Galantamine | nephrotoxic | training | COC1=C2O[C@H]3C[C@@H](O)C=C[C@]33CCN(C)CC(C=C1)=C23 |
| Tobramycin | nephrotoxic | training | NC[C@H]1O[C@H](O[C@@H]2[C@@H](N)C[C@@H](N)[C@H](O[C@H]3O[C@H](CO)[C@@H](O)[C@H](N)[C@H]3O)[C@H]2O)[C@H](N)C[C@@H]1O |
| Levocarnitine | nephrotoxic | training | C[N+](C)(C)C[C@H](O)CC([O-])=O |
| Butoconazole nitrate | non-nephrotoxic | training | ClC1=CC=C(CCC(CN2C=CN=C2)SC2=C(Cl)C=CC=C2Cl)C=C1 |
| Isoxsuprine | non-nephrotoxic | training | CC(COC1=CC=CC=C1)NC(C)C(O)C1=CC=C(O)C=C1 |
| Tazarotene | non-nephrotoxic | training | CCOC(=O)C1=CC=C(N=C1)C#CC1=CC=C2SCCC(C)(C)C2=C1 |
| DL-Thyroxine | non-nephrotoxic | training | NC(CC1=CC(I)=C(OC2=CC(I)=C(O)C(I)=C2)C(I)=C1)C(O)=O |
| Metoclopramide | non-nephrotoxic | training | CCN(CC)CCNC(=O)C1=C(OC)C=C(N)C(Cl)=C1 |
| Sertaconazole | non-nephrotoxic | training | ClC1=CC=C(C(CN2C=CN=C2)OCC2=CSC3=C(Cl)C=CC=C23)C(Cl)=C1 |
| valganciclovir | nephrotoxic | training | CC(C)[C@H](N)C(=O)OCC(CO)OCN1C=NC2=C1N=C(N)NC2=O |
| DSSTox_GSID_47840 | nephrotoxic | training | CCN(C)C(=O)OC1=CC(=CC=C1)[C@H](C)N(C)C |
| Cimetidine | nephrotoxic | training | CN=C(NCCSCC1=C(C)NC=N1)NC#N |
| didanosine | nephrotoxic | training | OC[C@@H]1CC[C@@H](O1)N1C=NC2=C1N=CNC2=O |
| Midodrine | non-nephrotoxic | training | COC1=CC=C(OC)C(=C1)C(O)CNC(=O)CN |
| [5-[(1S)-2-[(3R,6R,9R,11S,15R,20S,21R,24R,25R,26R)-3-[(1S)-3-Amino-1-hydroxy-3-oxopropyl]-11,20,21,25-tetrahydroxy-15-[(1R)-1-hydroxyethyl]-26-methyl-2,5,8,14,17,23-hexaoxo-18-[[4-[5-(4-pentoxyphenyl)-1,2-oxazol-3-yl]benzoyl]amino]-1,4,7,13,16,22-hexazatricyclo[22.3.0.09,13]heptacosan-6-yl]-1,2-dihydroxyethyl]-2-hydroxyphenyl] hydrogen sulfate | nephrotoxic | training | CCCCCOC1=CC=C(C=C1)C1=CC(=NO1)C1=CC=C(C=C1)C(=O)NC1C[C@H](O)[C@@H](O)NC(=O)[C@H]2[C@H](O)[C@H](C)CN2C(=O)[C@H](NC(=O)[C@H](NC(=O)[C@H]2C[C@H](O)CN2C(=O)[C@H](NC1=O)[C@@H](C)O)C(O)[C@@H](O)C1=CC=C(O)C(OS(O)(=O)=O)=C1)[C@@H](O)CC(N)=O |
| Ceftriaxone | nephrotoxic | training | CO\N=C(/C(=O)N[C@H]1[C@H]2SCC(CSC3=NC(=O)C(=O)NN3C)=C(N2C1=O)C(O)=O)C1=CSC(N)=N1 |
| Dexmedetomidine | nephrotoxic | training | C[C@H](C1=CN=CN1)C1=C(C)C(C)=CC=C1 |
| Fluticasone | non-nephrotoxic | training | C[C@@H]1C[C@H]2C3C[C@H](F)C4=CC(=O)C=C[C@]4(C)[C@@]3(F)[C@@H](O)C[C@]2(C)[C@@]1(O)C(=O)SCF |
| Estomycin | non-nephrotoxic | training | NCC1OC(OC2C(CO)OC(OC3C(O)C(N)CC(N)C3OC3OC(CO)C(O)C(O)C3N)C2O)C(N)C(O)C1O |
| Thiamine ion | non-nephrotoxic | training | CC1=C(CCO)SC=[N+]1CC1=C(N)N=C(C)N=C1 |
| Acipimox | non-nephrotoxic | training | CC1=[N+]([O-])C=C(N=C1)C(O)=O |
| Granisetron | non-nephrotoxic | training | CN1N=C(C(=O)NC2CC3CCCC(C2)N3C)C2=CC=CC=C12 |
| Desogestrel | non-nephrotoxic | training | CC[C@]12CC(=C)[C@H]3[C@@H](CCC4=CCCC[C@H]34)[C@@H]1CC[C@@]2(O)C#C |
| Butorphanol | non-nephrotoxic | training | OC1=CC2=C(CC3N(CC4CCC4)CCC22CCCCC32O)C=C1 |
| Ranitidine | non-nephrotoxic | training | CNC(NCCSCC1=CC=C(CN(C)C)O1)=C[N+]([O-])=O |
| Topiramate | nephrotoxic | training | CC1(C)O[C@@H]2CO[C@@]3(COS(N)(=O)=O)OC(C)(C)O[C@H]3[C@@H]2O1 |
| Dexpanthenol | non-nephrotoxic | training | CC(C)(CO)C(O)C(=O)NCCCO |
| Indinavir | nephrotoxic | training | CC(C)(C)NC(=O)[C@@H]1CN(CC2=CC=CN=C2)CCN1C[C@@H](O)C[C@@H](CC1=CC=CC=C1)C(=O)N[C@@H]1[C@H](O)CC2=C1C=CC=C2 |
| 2-Hydroxybutanedial | non-nephrotoxic | training | OC(CC=O)C=O |
| Atazanavir | nephrotoxic | training | COC(=O)N[C@H](C(=O)N[C@@H](CC1=CC=CC=C1)[C@@H](O)CN(CC1=CC=C(C=C1)C1=NC=CC=C1)NC(=O)[C@@H](NC(=O)OC)C(C)(C)C)C(C)(C)C |
| Capecitabine | nephrotoxic | training | CCCCCOC(=O)NC1=NC(=O)N(C=C1F)[C@@H]1O[C@H](C)[C@@H](O)[C@H]1O |
| Nalbuphine | non-nephrotoxic | training | OC1CCC2(O)C3CC4=CC=C(O)C5=C4C2(CCN3CC2CCC2)C1O5 |
| Naratriptan | nephrotoxic | training | CNS(=O)(=O)CCC1=CC=C2NC=C(C3CCN(C)CC3)C2=C1 |
| Dicloxacillin | nephrotoxic | training | CC1=C(C(=O)N[C@H]2[C@H]3SC(C)(C)[C@@H](N3C2=O)C(O)=O)C(=NO1)C1=C(Cl)C=CC=C1Cl |
| Piperacillin | nephrotoxic | training | CCN1CCN(C(=O)N[C@@H](C(=O)N[C@H]2[C@H]3SC(C)(C)[C@@H](N3C2=O)C(O)=O)C2=CC=CC=C2)C(=O)C1=O |
| VACV | nephrotoxic | training | CC(C)[C@H](N)C(=O)OCCOCN1C=NC2=C1N=C(N)NC2=O |
| Clopidogrel | nephrotoxic | training | COC(=O)[C@@H](N1CCC2=C(C1)C=CS2)C1=C(Cl)C=CC=C1 |
| Olmesartan | nephrotoxic | training | CCCC1=NC(=C(N1CC1=CC=C(C=C1)C1=C(C=CC=C1)C1=NNN=N1)C(O)=O)C(C)(C)O |
| Terazosin | non-nephrotoxic | training | COC1=C(OC)C=C2C(N)=NC(=NC2=C1)N1CCN(CC1)C(=O)C1CCCO1 |
| Nelarabine | nephrotoxic | training | COC1=C2N=CN([C@@H]3O[C@H](CO)[C@@H](O)[C@@H]3O)C2=NC(N)=N1 |
| Mepenzolate | non-nephrotoxic | training | C[N+]1(C)CCCC(C1)OC(=O)C(O)(C1=CC=CC=C1)C1=CC=CC=C1 |
| Dipyridamole | nephrotoxic | training | OCCN(CCO)C1=NC(N2CCCCC2)=C2N=C(N=C(N3CCCCC3)C2=N1)N(CCO)CCO |
| Ziconotide acetate | nephrotoxic | training | CSCCC1NC(=O)C(CC(C)C)NC(=O)C(CCCNC(N)=N)NC(=O)C(CO)NC(=O)C2CSSCC3NC(=O)C(CO)NC(=O)CNC(=O)C(NC(=O)C(CSSCC(N)C(=O)NC(CCCCN)C(=O)NCC(=O)NC(CCCCN)C(=O)NCC(=O)NC(C)C(=O)NC(CCCCN)C(=O)N2)NC(=O)C(CSSCC(NC(=O)C(CCCCN)NC(=O)CNC(=O)C(CO)NC(=O)C(CCCNC(N)=N)NC3=O)C(N)=O)NC(=O)C(CC(O)=O)NC(=O)C(CC2=CC=C(O)C=C2)NC1=O)C(C)O |
| Imatinib | nephrotoxic | training | CN1CCN(CC2=CC=C(C=C2)C(=O)NC2=CC=C(C)C(NC3=NC(=CC=N3)C3=CC=CN=C3)=C2)CC1 |
| Cysteamine | nephrotoxic | training | NCCS |
| Dimethyl sulfoxide | non-nephrotoxic | training | CS(C)=O |
| Flumethasone | non-nephrotoxic | training | CC1CC2C3CC(F)C4=CC(=O)C=CC4(C)C3(F)C(O)CC2(C)C1(O)C(=O)CO |
| Irbesartan | nephrotoxic | training | CCCCC1=NC2(CCCC2)C(=O)N1CC1=CC=C(C=C1)C1=C(C=CC=C1)C1=NNN=N1 |
| Penicillin | non-nephrotoxic | training | CC1(C)SC2C(NC(=O)CC3=CC=CC=C3)C(=O)N2C1C(O)=O |
| Fuzeon | nephrotoxic | training | CC[C@H](C)[C@H](NC(=O)[C@H](CC(C)C)NC(=O)[C@H](CO)NC(=O)[C@H](CC1=CN=CN1)NC(=O)[C@@H](NC(=O)[C@H](CC(C)C)NC(=O)[C@H](CO)NC(=O)[C@@H](NC(=O)[C@H](CC1=CC=C(O)C=C1)NC(C)=O)[C@@H](C)O)[C@@H](C)CC)C(=O)N[C@@H](CCC(O)=O)C(=O)N[C@@H](CCC(O)=O)C(=O)N[C@@H](CO)C(=O)N[C@@H](CCC(N)=O)C(=O)N[C@@H](CC(N)=O)C(=O)N[C@@H](CCC(N)=O)C(=O)N[C@@H](CCC(N)=O)C(=O)N[C@@H](CCC(O)=O)C(=O)N[C@@H](CCCCN)C(=O)N[C@@H](CC(N)=O)C(=O)N[C@@H](CCC(O)=O)C(=O)N[C@@H](CCC(N)=O)C(=O)N[C@@H](CCC(O)=O)C(=O)N[C@@H](CC(C)C)C(=O)N[C@@H](CC(C)C)C(=O)N[C@@H](CCC(O)=O)C(=O)N[C@@H](CC(C)C)C(=O)N[C@@H](CC(O)=O)C(=O)N[C@@H](CCCCN)C(=O)N[C@@H](CC1=CNC2=CC=CC=C12)C(=O)N[C@@H](C)C(=O)N[C@@H](CO)C(=O)N[C@@H](CC(C)C)C(=O)N[C@@H](CC1=CNC2=CC=CC=C12)C(=O)N[C@@H](CC(N)=O)C(=O)N[C@@H](CC1=CNC2=CC=CC=C12)C(=O)N[C@@H](CC1=CC=CC=C1)C(N)=O |
| Dtxsid10944385 | nephrotoxic | training | C(OC1=CC=C2OCOC2=C1)[C@@H]1CNCC[C@H]1C1=CC=CC=C1 |
| Cabergoline | non-nephrotoxic | training | CCNC(=O)N(CCCN(C)C)C(=O)C1CC2C(CC3=CNC4=CC=CC2=C34)N(CC=C)C1 |
| Adapalene | non-nephrotoxic | training | COC1=CC=C(C=C1C12CC3CC(CC(C3)C1)C2)C1=CC=C2C=C(C=CC2=C1)C(O)=O |
| Rilpivirine | nephrotoxic | training | CC1=CC(\C=C\C#N)=CC(C)=C1NC1=CC=NC(NC2=CC=C(C=C2)C#N)=N1 |
| Riluzole | nephrotoxic | training | NC1=NC2=CC=C(OC(F)(F)F)C=C2S1 |
| Ketoconazole | non-nephrotoxic | training | CC(=O)N1CCN(CC1)C1=CC=C(OCC2COC(CN3C=CN=C3)(O2)C2=CC=C(Cl)C=C2Cl)C=C1 |
| Mafenide | non-nephrotoxic | training | NCC1=CC=C(C=C1)S(N)(=O)=O |
| Interferon alfa-2B | nephrotoxic | training | CCCN(CCOC1=C(Cl)C=C(Cl)C=C1Cl)C(=O)N1C=CN=C1 |
| Saquinavir mesylate | nephrotoxic | training | CC(C)(C)NC(=O)[C@@H]1C[C@@H]2CCCC[C@@H]2CN1C[C@@H](O)[C@H](CC1=CC=CC=C1)NC(=O)[C@H](CC(N)=O)NC(=O)C1=CC=C2C=CC=CC2=N1 |
| Prostaglandin | non-nephrotoxic | training | CCCCCC(O)C=CC1C(O)CC(O)C1CC=CCCCC(O)=O |
| Propafenone | nephrotoxic | training | CCCNCC(O)COC1=C(C=CC=C1)C(=O)CCC1=CC=CC=C1 |
| Fosamprenavir calcium | nephrotoxic | training | CC(C)CN(C[C@@H](OP([O-])([O-])=O)[C@H](CC1=CC=CC=C1)NC(=O)O[C@H]1CCOC1)S(=O)(=O)C1=CC=C(N)C=C1 |
| Raloxifene | non-nephrotoxic | training | OC1=CC=C(C=C1)C1=C(C(=O)C2=CC=C(OCCN3CCCCC3)C=C2)C2=CC=C(O)C=C2S1 |
| Ramipril | nephrotoxic | training | CCOC(=O)[C@H](CCC1=CC=CC=C1)N[C@@H](C)C(=O)N1[C@H]2CCC[C@H]2C[C@H]1C(O)=O |
| Estra-1,3,5(10)-triene-3,17-diol | non-nephrotoxic | training | CC12CCC3C(CCC4=C3C=CC(O)=C4)C1CCC2O |
| Ritonavir | nephrotoxic | training | CC(C)[C@H](NC(=O)N(C)CC1=CSC(=N1)C(C)C)C(=O)N[C@H](C[C@H](O)[C@H](CC1=CC=CC=C1)NC(=O)OCC1=CN=CS1)CC1=CC=CC=C1 |
| Diflunisal | nephrotoxic | training | OC(=O)C1=CC(=CC=C1O)C1=CC=C(F)C=C1F |
| Panhematin | nephrotoxic | training | CC1=C(CCC(O)=O)/C2=C/C3=C(CCC(O)=O)C(C)=C([N-]3)\C=C3/N=C(/C=C4\N=C(\C=C\1/[N-]\2)C(C)=C4C=C)C(C)=C3C=C |
| Telithromycin | non-nephrotoxic | training | CC[C@H]1OC(=O)[C@H](C)C(=O)[C@H](C)[C@@H](O[C@@H]2O[C@H](C)C[C@@H]([C@H]2O)N(C)C)[C@@](C)(C[C@@H](C)C(=O)[C@@H](C)[C@H]2N(CCCCN3C=NC(=C3)C3=CC=CN=C3)C(=O)O[C@]12C)OC |
| Phenazopyridine | non-nephrotoxic | training | NC1=CC=C(N=NC2=CC=CC=C2)C(N)=N1 |
| Gamma-aminobutyric acid | non-nephrotoxic | training | NCCCC(O)=O |
| Beta-Methasone alcohol | non-nephrotoxic | training | CC1CC2C3CCC4=CC(=O)C=CC4(C)C3(F)C(O)CC2(C)C1(O)C(=O)CO |
| Kinetin | non-nephrotoxic | training | C(NC1=C2NC=NC2=NC=N1)C1=CC=CO1 |
| Varenicline | nephrotoxic | training | C1C2CNCC1C1=C2C=C2N=CC=NC2=C1 |
| Desmopressin | non-nephrotoxic | training | NC(=O)CCC1NC(=O)C(CC2=CC=CC=C2)NC(=O)C(CC2=CC=C(O)C=C2)NC(=O)CCSSCC(NC(=O)C(CC(N)=O)NC1=O)C(=O)N1CCCC1C(=O)NC(CCCN=C(N)N)C(=O)NCC(N)=O |
| Cyclophosphamide | nephrotoxic | training | ClCCN(CCCl)P1(=O)NCCCO1 |
| Minoxidil | non-nephrotoxic | training | NC1=CC(=NC(=N)N1O)N1CCCCC1 |
| Teniposide | nephrotoxic | training | COC1=CC(=CC(OC)=C1O)[C@H]1[C@@H]2[C@H](COC2=O)[C@H](O[C@@H]2O[C@@H]3CO[C@H](O[C@H]3[C@H](O)[C@H]2O)C2=CC=CS2)C2=C1C=C1OCOC1=C2 |
| Vildagliptin | non-nephrotoxic | training | OC12CC3CC(C1)CC(C3)(C2)NCC(=O)N1CCCC1C#N |
| Nexium | nephrotoxic | training | COC1=CC=C2[N-]C(=NC2=C1)[S@@](=O)CC1=C(C)C(OC)=C(C)C=N1 |
| Naproxen | nephrotoxic | training | COC1=CC2=CC=C(C=C2C=C1)[C@H](C)C(O)=O |
| Phosphonatoformate | nephrotoxic | training | [O-]C(=O)P([O-])([O-])=O |
| deferasirox | nephrotoxic | training | OC(=O)C1=CC=C(C=C1)N1N=C(N=C1C1=C(O)C=CC=C1)C1=C(O)C=CC=C1 |
| Pentazocine | non-nephrotoxic | training | CC1C2CC3=C(C=C(O)C=C3)C1(C)CCN2CC=C(C)C |
| Sulfadiazine | non-nephrotoxic | training | NC1=CC=C(C=C1)S(=O)(=O)NC1=NC=CC=N1 |
| Lomefloxacin | nephrotoxic | training | CCN1C=C(C(O)=O)C(=O)C2=C1C(F)=C(N1CCNC(C)C1)C(F)=C2 |
| Isoprenaline | non-nephrotoxic | training | CC(C)NCC(O)C1=CC=C(O)C(O)=C1 |
| Fondaparinux sodium | non-nephrotoxic | training | CO[C@H]1O[C@H](COS([O-])(=O)=O)[C@@H](O[C@@H]2O[C@H]([C@@H](O[C@H]3O[C@H](COS([O-])(=O)=O)C(O[C@@H]4O[C@@H]([C@@H](O[C@H]5O[C@H](COS([O-])(=O)=O)[C@@H](O)[C@H](O)[C@H]5NS([O-])(=O)=O)[C@H](O)[C@H]4O)C([O-])=O)[C@H](OS([O-])(=O)=O)[C@H]3NS([O-])(=O)=O)[C@H](O)[C@H]2OS([O-])(=O)=O)C([O-])=O)[C@H](O)[C@H]1NS([O-])(=O)=O |
| Dutasteride | non-nephrotoxic | training | C[C@]12CCC3[C@@H](CC[C@H]4NC(=O)C=C[C@]34C)[C@@H]1CC[C@@H]2C(=O)NC1=CC(=CC=C1C(F)(F)F)C(F)(F)F |
| Ceftibuten dihydrate | nephrotoxic | training | NC1=NC(=CS1)C(=C\CC(O)=O)\C(=O)N[C@H]1[C@H]2SCC=C(N2C1=O)C(O)=O |
| 11,17,21-Trihydroxypregn-4-ene-3,20-dione | non-nephrotoxic | training | CC12CC(O)C3C(CCC4=CC(=O)CCC34C)C1CCC2(O)C(=O)CO |
| Tocophersolan | non-nephrotoxic | training | CC(C)CCCC(C)CCCC(C)CCCC1(C)CCC2=C(C)C(OC(=O)CCC(=O)OCCO)=C(C)C(C)=C2O1 |
| Celiprolol | non-nephrotoxic | training | CCN(CC)C(=O)NC1=CC=C(OCC(O)CNC(C)(C)C)C(=C1)C(C)=O |
| Gemeprost | non-nephrotoxic | training | CCCCC(C)(C)C(O)C=CC1C(O)CC(=O)C1CCCCC=CC(=O)OC |
| Ivabradine | non-nephrotoxic | training | COC1=CC2=C(C=C1OC)[C@@H](CN(C)CCCN1CCC3=C(CC1=O)C=C(OC)C(OC)=C3)C2 |
| (-)-Methyldopa | non-nephrotoxic | training | CC(N)(CC1=CC=C(O)C(O)=C1)C(O)=O |
| Thalidomide | nephrotoxic | training | O=C1N(C2CCC(=O)NC2=O)C(=O)C2=C1C=CC=C2 |
| Fuyosyosin | non-nephrotoxic | training | CN1CCCC1CCOC(C)(C1=CC=CC=C1)C1=CC=C(Cl)C=C1 |
| Gonadorelin | non-nephrotoxic | training | CC(C)CC(NC(=O)CNC(=O)C(CC1=CC=C(O)C=C1)NC(=O)C(CO)NC(=O)C(CC1=CNC2=CC=CC=C12)NC(=O)C(CC1=CN=CN1)NC(=O)C1CCC(=O)N1)C(=O)NC(CCCN=C(N)N)C(=O)N1CCCC1C(=O)NCC(N)=O |
| Protirelin | non-nephrotoxic | training | NC(=O)C1CCCN1C(=O)C(CC1=CN=CN1)NC(=O)C1CCC(=O)N1 |
| acyclovir | nephrotoxic | training | NC1=NC2=C(N=CN2COCCO)C(=O)N1 |
| Cyproterone | non-nephrotoxic | training | CC(=O)C1(O)CCC2C3C=C(Cl)C4=CC(=O)C5CC5C4(C)C3CCC12C |
| Deptomycin | nephrotoxic | training | CCCCCCCCCC(=O)NC(CC1=CNC2=CC=CC=C12)C(=O)NC(CC(N)=O)C(=O)NC(CC(O)=O)C(=O)NC1C(C)OC(=O)C(CC(=O)C2=C(N)C=CC=C2)NC(=O)C(NC(=O)C(CO)NC(=O)CNC(=O)C(CC(O)=O)NC(=O)C(C)NC(=O)C(CC(O)=O)NC(=O)C(CCCN)NC(=O)CNC1=O)C(C)CC(O)=O |
| Cefuroxime | nephrotoxic | training | CO\N=C(/C(=O)N[C@H]1[C@H]2SCC(COC(N)=O)=C(N2C1=O)C(O)=O)C1=CC=CO1 |
| Ceftazidime | nephrotoxic | training | CC(C)(O\N=C(/C(=O)N[C@H]1[C@H]2SCC(C[N+]3=CC=CC=C3)=C(N2C1=O)C([O-])=O)C1=CSC(N)=N1)C(O)=O |
| Ridauran | nephrotoxic | training | CC(=O)OC[C@H]1O[C@@H]([S-])[C@H](OC(C)=O)[C@@H](OC(C)=O)[C@@H]1OC(C)=O |
| olsalazine | nephrotoxic | training | OC(=O)C1=CC(=CC=C1O)N=NC1=CC=C(O)C(=C1)C(O)=O |
| Carisoprodol | non-nephrotoxic | training | CCCC(C)(COC(N)=O)COC(=O)NC(C)C |
| Orlistat | nephrotoxic | training | CCCCCCCCCCC[C@@H](C[C@@H]1OC(=O)[C@H]1CCCCCC)OC(=O)[C@H](CC(C)C)NC=O |
| (r)-Omeprazole | nephrotoxic | training | COC1=CC=C2N=C(NC2=C1)[S@](=O)CC1=C(C)C(OC)=C(C)C=N1 |
| Cladribine | nephrotoxic | training | NC1=C2N=CN([C@H]3C[C@H](O)[C@@H](CO)O3)C2=NC(Cl)=N1 |
| Propagermanium | nephrotoxic | training | OC(=O)CC[Ge](=O)O[Ge](=O)CCC(O)=O |
| Doripenem | non-nephrotoxic | training | C[C@@H](O)[C@@H]1[C@H]2[C@@H](C)C(S[C@@H]3CN[C@H](CNS(N)(=O)=O)C3)=C(N2C1=O)C(O)=O |
| 2-Bromo-alpha-ergocryptine | non-nephrotoxic | training | CC(C)CC1N2C(=O)C(NC(=O)C3CN(C)C4CC5=C(Br)NC6=CC=CC(=C56)C4=C3)(OC2(O)C2CCCN2C1=O)C(C)C |
| Cefaclor hydrate | nephrotoxic | training | N[C@@H](C(=O)N[C@H]1[C@H]2SCC(Cl)=C(N2C1=O)C(O)=O)C1=CC=CC=C1 |
| Icatibant acetate | non-nephrotoxic | training | N[C@H](CCCN=C(N)N)C(=O)NC(CCCN=C(N)N)C(=O)N1CCCC1C(=O)N1C[C@H](O)CC1C(=O)NCC(=O)N[C@@H](CC1=CC=CS1)C(=O)N[C@@H](CO)C(=O)N1CC2=C(CC1C(=O)N1[C@H]3CCCC[C@H]3CC1C(=O)N[C@@H](CCCN=C(N)N)C(O)=O)C=CC=C2 |
| Rimantadine | non-nephrotoxic | training | CC(N)C12CC3CC(CC(C3)C1)C2 |
| Bexarotene | nephrotoxic | training | CC1=C(C=C2C(=C1)C(C)(C)CCC2(C)C)C(=C)C1=CC=C(C=C1)C(O)=O |
| Tolvaptan | nephrotoxic | training | CC1=CC(NC(=O)C2=C(C)C=CC=C2)=CC=C1C(=O)N1CCCC(O)C2=C1C=CC(Cl)=C2 |
| Alosetron | non-nephrotoxic | training | CN1C2=C(C3=CC=CC=C13)C(=O)N(CC1=C(C)NC=N1)CC2 |
| Eflornithine | non-nephrotoxic | training | NCCCC(N)(C(F)F)C(O)=O |
| Sparfloxacin | nephrotoxic | training | C[C@H]1CN(C[C@@H](C)N1)C1=C(F)C2=C(C(N)=C1F)C(=O)C(=CN2C1CC1)C(O)=O |
| Ofloxacin | nephrotoxic | training | CC1COC2=C(N3CCN(C)CC3)C(F)=CC3=C2N1C=C(C(O)=O)C3=O |
| Metformin | non-nephrotoxic | training | CN(C)C(=N)N=C(N)N |
| Mexiletine | non-nephrotoxic | training | CC(N)COC1=C(C)C=CC=C1C |
| Sitagliptin | nephrotoxic | training | N[C@@H](CC(=O)N1CCN2C(C1)=NN=C2C(F)(F)F)CC1=C(F)C=C(F)C(F)=C1 |
| Simendan | non-nephrotoxic | training | CC1CC(=O)NN=C1C1=CC=C(NN=C(C#N)C#N)C=C1 |
| Pyridoxine | non-nephrotoxic | training | CC1=C(O)C(CO)=C(CO)C=N1 |
| Isradipine | non-nephrotoxic | training | COC(=O)C1=C(C)NC(C)=C(C1C1=CC=CC2=NON=C12)C(=O)OC(C)C |
| Mupirocin | non-nephrotoxic | training | CC(O)C(C)C1OC1CC1COC(CC(C)=CC(=O)OCCCCCCCCC(O)=O)C(O)C1O |
| Gemifloxacin | nephrotoxic | training | CO\N=C1/CN(CC1CN)C1=NC2=C(C=C1F)C(=O)C(=CN2C1CC1)C(O)=O |
| Oxandrolone | non-nephrotoxic | training | C[C@]1(O)CC[C@H]2[C@@H]3CC[C@H]4CC(=O)OC[C@]4(C)[C@H]3CC[C@]12C |
| (2R)-2-Hydroxybutanedial | nephrotoxic | training | O[C@H](CC=O)C=O |
| Arginine | non-nephrotoxic | training | NC(CCCN=C(N)N)C(O)=O |
| Norfloxacin | nephrotoxic | training | CCN1C=C(C(O)=O)C(=O)C2=C1C=C(N1CCNCC1)C(F)=C2 |
| Beconase AQ | non-nephrotoxic | training | CCC(=O)OCC(=O)C1(OC(=O)CC)C(C)CC2C3CCC4=CC(=O)C=CC4(C)C3(Cl)C(O)CC12C |
| Glycerin | non-nephrotoxic | training | OCC(O)CO |
| 6-mercaptopurine | non-nephrotoxic | training | S=C1N=CNC2=C1NC=N2 |
| Tinidazole | non-nephrotoxic | training | CCS(=O)(=O)CCN1C(C)=NC=C1[N+]([O-])=O |
| Fenoldopam | non-nephrotoxic | training | OC1=CC=C(C=C1)C1C[NH2+]CCC2=C1C=C(O)C(O)=C2Cl |
| Acamprosate | nephrotoxic | training | CC(=O)NCCCS(O)(=O)=O |
| Rathyronine | non-nephrotoxic | training | NC(CC1=CC(I)=C(OC2=CC=C(O)C(I)=C2)C(I)=C1)C(O)=O |
| Buscopan | non-nephrotoxic | training | CCCC[N+]1(C)C2CC(CC1C1OC21)OC(=O)C(CO)C1=CC=CC=C1 |
| Kivexa | nephrotoxic | training | NC1=NC(NC2CC2)=C2N=CN([C@@H]3C[C@H](CO)C=C3)C2=N1 |
| Tolbutamide | non-nephrotoxic | training | CCCCNC(=O)NS(=O)(=O)C1=CC=C(C)C=C1 |
| Rasagiline mesylate | nephrotoxic | training | C#CCN[C@@H]1CCC2=C1C=CC=C2 |
| Tacrolimus | nephrotoxic | training | CO[C@@H]1C[C@@H](CC[C@H]1O)\C=C(/C)[C@H]1OC(=O)[C@@H]2CCCCN2C(=O)C(=O)[C@]2(O)O[C@@H]([C@H](C[C@H]2C)OC)[C@H](C[C@@H](C)C\C(C)=C/[C@@H](CC=C)C(=O)C[C@H](O)[C@H]1C)OC |
| Palonosetron | non-nephrotoxic | training | O=C1N(C[C@@H]2CCCC3=C2C1=CC=C3)[C@@H]1CN2CCC1CC2 |
| Rimexolone | non-nephrotoxic | training | CCC(=O)[C@@]1(C)[C@H](C)CC2C3CCC4=CC(=O)C=C[C@]4(C)C3[C@@H](O)C[C@]12C |
| Pregabalin | nephrotoxic | training | CC(C)C[C@H](CN)CC(O)=O |
| Clobetasone butyrate | non-nephrotoxic | training | CCCC(=O)OC1(C(C)CC2C3CCC4=CC(=O)C=CC4(C)C3(F)C(=O)CC12C)C(=O)CCl |
| Miglitol | non-nephrotoxic | training | OCCN1CC(O)C(O)C(O)C1CO |
| Fluoxetine | nephrotoxic | training | CNCCC(OC1=CC=C(C=C1)C(F)(F)F)C1=CC=CC=C1 |
| Sumatriptan | nephrotoxic | training | CNS(=O)(=O)CC1=CC=C2NC=C(CCN(C)C)C2=C1 |
| Tretinoin | nephrotoxic | training | C\C(\C=C\C1=C(C)CCCC1(C)C)=C/C=C/C(/C)=C/C(O)=O |
| Halodrin (Salt/Mix) | non-nephrotoxic | training | CC1(O)CCC2C3CCC4=CC(=O)CCC4(C)C3(F)C(O)CC12C |
| Clomipramine | nephrotoxic | training | CN(C)CCCN1C2=C(CCC3=C1C=C(Cl)C=C3)C=CC=C2 |
| Ibuprofen | nephrotoxic | training | CC(C)CC1=CC=C(C=C1)C(C)C(O)=O |
| Voriconazole | nephrotoxic | training | C[C@@H](C1=C(F)C=NC=N1)[C@](O)(CN1C=NC=N1)C1=CC=C(F)C=C1F |
| Nimodipine | non-nephrotoxic | training | COCCOC(=O)C1=C(C)NC(C)=C(C1C1=CC=CC(=C1)[N+]([O-])=O)C(=O)OC(C)C |
| Griseofulvin, (+)- | non-nephrotoxic | training | COC1=CC(OC)=C(Cl)C2=C1C(=O)C1(O2)C(C)CC(=O)C=C1OC |
| Tranexamic acid | nephrotoxic | training | NCC1CCC(CC1)C(O)=O |
| 2-[4-[(E)-2-Chloro-1,2-diphenylethenyl]phenoxy]ethyl-diethylazanium | nephrotoxic | training | CC[NH+](CC)CCOC1=CC=C(C=C1)C(=C(\Cl)C1=CC=CC=C1)\C1=CC=CC=C1 |
| Mitoxantrone | nephrotoxic | training | OCCNCCNC1=CC=C(NCCNCCO)C2=C1C(=O)C1=C(C(O)=CC=C1O)C2=O |
| Suprofen | non-nephrotoxic | training | CC(C(O)=O)C1=CC=C(C=C1)C(=O)C1=CC=CS1 |
| Nisoldipine | non-nephrotoxic | training | COC(=O)C1=C(C)NC(C)=C(C1C1=C(C=CC=C1)[N+]([O-])=O)C(=O)OCC(C)C |
| Selegiline | nephrotoxic | training | C[C@H](CC1=CC=CC=C1)N(C)CC#C |
| Pomalidomide | nephrotoxic | training | NC1=CC=CC2=C1C(=O)N(C1CCC(=O)NC1=O)C2=O |
| Diethylpropion | non-nephrotoxic | training | CCN(CC)C(C)C(=O)C1=CC=CC=C1 |
| Solifenacin hydrochloride | nephrotoxic | training | O=C(O[C@H]1CN2CCC1CC2)N1CCC2=C(C=CC=C2)[C@@H]1C1=CC=CC=C1 |
| Remifentanil hydrochloride | nephrotoxic | training | CCC(=O)N(C1=CC=CC=C1)C1(CCN(CCC(=O)OC)CC1)C(=O)OC |
| Aminoglutethimide | non-nephrotoxic | training | CCC1(CCC(=O)NC1=O)C1=CC=C(N)C=C1 |
| Neo-Oxylone (Salt/Mix) | non-nephrotoxic | training | CC1CC2C3CCC(O)(C(C)=O)C3(C)CC(O)C2(F)C2(C)C=CC(=O)C=C12 |
| Verapamil | non-nephrotoxic | training | COC1=CC=C(CCN(C)CCCC(C#N)(C(C)C)C2=CC=C(OC)C(OC)=C2)C=C1OC |
| Oxprenolol | non-nephrotoxic | training | CC(C)NCC(O)COC1=C(OCC=C)C=CC=C1 |
| Pentosan polysulfate | non-nephrotoxic | training | O[C@@H]1CO[C@@H](O[C@@H]2CO[C@@H](O)[C@H](OS(O)(=O)=O)[C@H]2OS(O)(=O)=O)[C@H](OS(O)(=O)=O)[C@H]1OS(O)(=O)=O |
| Hydroxyprogesterone | non-nephrotoxic | training | CC(=O)[C@@]1(O)CC[C@H]2[C@@H]3CCC4=CC(=O)CC[C@]4(C)[C@H]3CC[C@]12C |
| Ketamine | non-nephrotoxic | training | CNC1(CCCCC1=O)C1=C(Cl)C=CC=C1 |
| Flucytosine | non-nephrotoxic | training | NC1=C(F)C=NC(=O)N1 |
| Salicylic acid | non-nephrotoxic | training | OC(=O)C1=C(O)C=CC=C1 |
| Clarithromycin | nephrotoxic | training | CC[C@H]1OC(=O)[C@H](C)[C@@H](O[C@H]2C[C@@](C)(OC)[C@@H](O)[C@H](C)O2)[C@H](C)[C@@H](O[C@@H]2O[C@H](C)C[C@@H]([C@H]2O)N(C)C)[C@@](C)(C[C@@H](C)C(=O)[C@H](C)[C@@H](O)[C@]1(C)O)OC |
| Azithromycin | nephrotoxic | training | CC[C@H]1OC(=O)[C@H](C)[C@@H](O[C@H]2C[C@@](C)(OC)[C@@H](O)[C@H](C)O2)[C@H](C)[C@@H](O[C@@H]2O[C@H](C)C[C@@H]([C@H]2O)N(C)C)[C@](C)(O)C[C@@H](C)CN(C)[C@H](C)[C@@H](O)[C@]1(C)O |
| Atorvastatin calcium | nephrotoxic | training | CC(C)C1=C(C(=O)NC2=CC=CC=C2)C(=C(N1CC[C@@H](O)C[C@@H](O)CC([O-])=O)C1=CC=C(F)C=C1)C1=CC=CC=C1 |
| Vandetanib | nephrotoxic | training | COC1=C(OCC2CCN(C)CC2)C=C2N=CN=C(NC3=CC=C(Br)C=C3F)C2=C1 |
| Docosanol | non-nephrotoxic | training | CCCCCCCCCCCCCCCCCCCCCCO |
| Herplex | non-nephrotoxic | training | OCC1OC(CC1O)N1C=C(I)C(=O)NC1=O |
| Ampicillin | non-nephrotoxic | training | CC1(C)SC2C(NC(=O)C(N)C3=CC=CC=C3)C(=O)N2C1C([O-])=O |
| Rocuronium | non-nephrotoxic | training | CC(=O)O[C@H]1[C@H](CC2C3CCC4C[C@H](O)[C@H](C[C@]4(C)C3CC[C@]12C)N1CCOCC1)[N+]1(CC=C)CCCC1 |
| Nicotinate | non-nephrotoxic | training | [O-]C(=O)C1=CC=CN=C1 |
| Tiagabine | nephrotoxic | training | CC1=C(SC=C1)C(=CCCN1CCC[C@H](C1)C(O)=O)C1=C(C)C=CS1 |
| Lamotrigine | nephrotoxic | training | NC1=NN=C(C(N)=N1)C1=CC=CC(Cl)=C1Cl |
| Conivaptan | non-nephrotoxic | training | CC1=NC2=C(CCN(C(=O)C3=CC=C(NC(=O)C4=C(C=CC=C4)C4=CC=CC=C4)C=C3)C3=C2C=CC=C3)N1 |
| Perhexiline | non-nephrotoxic | training | C(C(C1CCCCC1)C1CCCCC1)C1CCCCN1 |
| Chloroprocaine | non-nephrotoxic | training | CCN(CC)CCOC(=O)C1=CC=C(N)C=C1Cl |
| Mebeverine | non-nephrotoxic | training | CCN(CCCCOC(=O)C1=CC=C(OC)C(OC)=C1)C(C)CC1=CC=C(OC)C=C1 |
| Amlexanox | non-nephrotoxic | training | CC(C)C1=CC=C2OC3=C(C=C(C(O)=O)C(N)=N3)C(=O)C2=C1 |
| Nicorandil | non-nephrotoxic | training | [O-][N+](=O)OCCNC(=O)C1=CC=CN=C1 |
| IRINOTECAN HYDROCHLORIDE Trihydrate | nephrotoxic | training | CCC1=C2CN3C(=CC4=C(COC(=O)[C@]4(O)CC)C3=O)C2=NC2=CC=C(OC(=O)N3CCC(CC3)N3CCCCC3)C=C12 |
| Bramazil | non-nephrotoxic | training | CN(CC=CC#CC(C)(C)C)CC1=C2C=CC=CC2=CC=C1 |
| Nadolol | non-nephrotoxic | training | CC(C)(C)NCC(O)COC1=C2CC(O)C(O)CC2=CC=C1 |
| Carglumic acid | non-nephrotoxic | training | NC(=O)N[C@@H](CCC(O)=O)C(O)=O |
| Gemcitabine hydrochloride | nephrotoxic | training | NC1=NC(=O)N(C=C1)[C@@H]1O[C@H](CO)[C@@H](O)C1(F)F |
| Emtricitabine | nephrotoxic | training | NC1=NC(=O)N(C=C1F)[C@@H]1CS[C@H](CO)O1 |
| Bile salt | non-nephrotoxic | training | CC(CCC(O)=O)C1CCC2C3C(O)CC4CC(O)CCC4(C)C3CC(O)C12C |
| Everolimus | nephrotoxic | training | CO[C@@H]1C[C@H](C[C@@H](C)[C@@H]2CC(=O)[C@H](C)\C=C(C)/[C@@H](O)[C@@H](OC)C(=O)[C@H](C)C[C@H](C)\C=C\C=C\C=C(C)\[C@H](C[C@@H]3CC[C@@H](C)[C@@](O)(O3)C(=O)C(=O)N3CCCC[C@H]3C(=O)O2)OC)CC[C@H]1OCCO |
| Lactic acid | non-nephrotoxic | training | CC(O)C(O)=O |
| Terlipressin | non-nephrotoxic | training | NCCCC[C@H](NC(=O)[C@@H]1CCCN1C(=O)[C@@H]1CSSC[C@H](NC(=O)CNC(=O)CNC(=O)CN)C(=O)N[C@@H](CC2=CC=C(O)C=C2)C(=O)N[C@@H](CC2=CC=CC=C2)C(=O)N[C@@H](CCC(N)=O)C(=O)N[C@@H](CC(N)=O)C(=O)N1)C(=O)NCC(N)=O |
| Phenytoin | nephrotoxic | training | O=C1NC(=O)C(N1)(C1=CC=CC=C1)C1=CC=CC=C1 |
| Lisinopril dihydrate | nephrotoxic | training | NCCCC[C@H](N[C@@H](CCC1=CC=CC=C1)C(O)=O)C(=O)N1CCC[C@H]1C(O)=O |
| Glipizide | non-nephrotoxic | training | CC1=NC=C(N=C1)C(=O)NCCC1=CC=C(C=C1)S(=O)(=O)NC(=O)NC1CCCCC1 |
| Carteolol | non-nephrotoxic | training | CC(C)(C)NCC(O)COC1=C2CCC(=O)NC2=CC=C1 |
| Ibandronic Acid | nephrotoxic | training | CCCCCN(C)CCC(O)(P(O)(O)=O)P(O)(O)=O |
| Afatinib | nephrotoxic | training | CN(C)C\C=C\C(=O)NC1=C(O[C@H]2CCOC2)C=C2N=CN=C(NC3=CC=C(F)C(Cl)=C3)C2=C1 |
| Scopolamine | non-nephrotoxic | training | CN1C2CC(CC1C1OC21)OC(=O)C(CO)C1=CC=CC=C1 |
| Pergolide | nephrotoxic | training | CCCN1C[C@H](CSC)C[C@H]2[C@H]1CC1=CNC3=CC=CC2=C13 |
| Mifepristone | non-nephrotoxic | training | CC#CC1(O)CCC2C3CCC4=CC(=O)CCC4=C3C(CC12C)C1=CC=C(C=C1)N(C)C |
| Metyrosine | non-nephrotoxic | training | CC(N)(CC1=CC=C(O)C=C1)C(O)=O |
| Bretylium | non-nephrotoxic | training | CC[N+](C)(C)CC1=C(Br)C=CC=C1 |
| Methocarbamol | non-nephrotoxic | training | COC1=C(OCC(O)COC(N)=O)C=CC=C1 |
| Nicardipine | non-nephrotoxic | training | COC(=O)C1=C(C)NC(C)=C(C1C1=CC=CC(=C1)[N+]([O-])=O)C(=O)OCC[NH+](C)CC1=CC=CC=C1 |
| Amiloride hydrochloride | non-nephrotoxic | training | NC(N)=NC(=O)C1=NC(Cl)=C(N)N=C1N |
| pemetrexed | nephrotoxic | training | NC1=NC2=C(C(CCC3=CC=C(C=C3)C(=O)N[C@@H](CCC(O)=O)C(O)=O)=CN2)C(=O)N1 |
| Lurasidone | nephrotoxic | training | O=C1[C@H]2[C@@H]3CC[C@@H](C3)[C@H]2C(=O)N1C[C@@H]1CCCC[C@H]1CN1CCN(CC1)C1=NSC2=CC=CC=C12 |
| Felodipine | non-nephrotoxic | training | CCOC(=O)C1=C(C)NC(C)=C(C1C1=CC=CC(Cl)=C1Cl)C(=O)OC |
| Cidofovir | nephrotoxic | training | NC1=NC(=O)N(C[C@@H](CO)OCP(O)(O)=O)C=C1 |
| Bortezomib | nephrotoxic | training | CC(C)C[C@H](NC(=O)[C@H](CC1=CC=CC=C1)NC(=O)C1=CN=CC=N1)B(O)O |
| Sirolimus | nephrotoxic | training | CO[C@@H]1C[C@H](C[C@@H](C)[C@@H]2CC(=O)[C@H](C)\C=C(C)/[C@@H](O)[C@@H](OC)C(=O)[C@H](C)C[C@H](C)\C=C\C=C\C=C(C)\[C@H](C[C@@H]3CC[C@@H](C)[C@@](O)(O3)C(=O)C(=O)N3CCCC[C@H]3C(=O)O2)OC)CC[C@H]1O |
| Tetracaine | non-nephrotoxic | training | CCCCNC1=CC=C(C=C1)C(=O)OCCN(C)C |
| Tolcapone | nephrotoxic | training | CC1=CC=C(C=C1)C(=O)C1=CC(O)=C(O)C(=C1)[N+]([O-])=O |
| Nefazodone | nephrotoxic | training | CCC1=NN(CCCN2CCN(CC2)C2=CC=CC(Cl)=C2)C(=O)N1CCOC1=CC=CC=C1 |
| Dasatinib | nephrotoxic | training | CC1=NC(=CC(NC2=NC=C(S2)C(=O)NC2=C(Cl)C=CC=C2C)=N1)N1CCN(CCO)CC1 |
| 2-[2-[4-[(R)-(4-Chlorophenyl)-phenylmethyl]piperazine-1,4-diium-1-yl]ethoxy]acetate | nephrotoxic | training | [O-]C(=O)COCC[NH+]1CC[NH+](CC1)[C@H](C1=CC=CC=C1)C1=CC=C(Cl)C=C1 |
| balsalazide | nephrotoxic | training | OC(=O)CCNC(=O)C1=CC=C(C=C1)N=NC1=CC=C(O)C(=C1)C(O)=O |
| Venlafaxine | nephrotoxic | training | COC1=CC=C(C=C1)C(CN(C)C)C1(O)CCCCC1 |
| Phenoxybenzamine | non-nephrotoxic | training | CC(COC1=CC=CC=C1)N(CCCl)CC1=CC=CC=C1 |
| Nebivolol | nephrotoxic | training | OC(CNCC(O)C1CCC2=C(O1)C=CC(F)=C2)C1CCC2=C(O1)C=CC(F)=C2 |
| Bacitracin A | non-nephrotoxic | training | CCC(C)C(N)C1=NC(CS1)C(=O)NC(CC(C)C)C(=O)NC(CCC(O)=O)C(=O)NC(C(C)CC)C(=O)NC1CCCCNC(=O)C(CC(N)=O)NC(=O)C(CC(O)=O)NC(=O)C(CC2=CN=CN2)NC(=O)C(CC2=CC=CC=C2)NC(=O)C(NC(=O)C(CCCN)NC1=O)C(C)CC |
| Simvastatin | nephrotoxic | training | CCC(C)(C)C(=O)O[C@H]1C[C@@H](C)C=C2C=C[C@H](C)[C@H](CC[C@@H]3C[C@@H](O)CC(=O)O3)[C@@H]12 |
| Ifosfamide | nephrotoxic | training | ClCCNP1(=O)OCCCN1CCCl |
| Lansoprazole | nephrotoxic | training | CC1=C(CS(=O)C2=NC3=CC=CC=C3N2)N=CC=C1OCC(F)(F)F |
| Mykostin | non-nephrotoxic | training | CC(C)C(C)C=CC(C)C1CCC2C(CCCC12C)=CC=C1CC(O)CCC1=C |
| Phylloquinone | non-nephrotoxic | training | CC(C)CCCC(C)CCCC(C)CCCC(C)=CCC1=C(C)C(=O)C2=C(C=CC=C2)C1=O |
| Zonisamide | nephrotoxic | training | NS(=O)(=O)CC1=NOC2=CC=CC=C12 |
| Diphenhydramine | non-nephrotoxic | training | CN(C)CCOC(C1=CC=CC=C1)C1=CC=CC=C1 |
| Adefovir dipivoxil | nephrotoxic | training | CC(C)(C)C(=O)OCOP(=O)(COCCN1C=NC2=C(N)N=CN=C12)OCOC(=O)C(C)(C)C |
| Dicyclomine | non-nephrotoxic | training | CCN(CC)CCOC(=O)C1(CCCCC1)C1CCCCC1 |
| Deferiprone | nephrotoxic | training | CN1C=CC(=O)C(O)=C1C |
| [(5S)-Spiro[8-azoniabicyclo[3.2.1]octane-8,1'-azolidin-1-ium]-3-yl] 2-hydroxy-2,2-diphenylacetate;chloride | nephrotoxic | training | OC(C(=O)OC1CC2CC[C@@H](C1)[N+]21CCCC1)(C1=CC=CC=C1)C1=CC=CC=C1 |
| Docetaxolum | nephrotoxic | training | CC(=O)O[C@@]12CO[C@@H]1C[C@H](O)[C@]1(C)[C@@H]2[C@H](OC(=O)C2=CC=CC=C2)[C@]2(O)C[C@H](OC(=O)C(O)[C@@H](NC(=O)OC(C)(C)C)C3=CC=CC=C3)C(C)=C([C@@H](O)C1=O)C2(C)C |
| Duloxetine hydrochloride | nephrotoxic | training | CNCC[C@H](OC1=C2C=CC=CC2=CC=C1)C1=CC=CS1 |
| olanzapine | nephrotoxic | training | CN1CCN(CC1)C1=NC2=C(NC3=C1C=C(C)S3)C=CC=C2 |
| Obeticholic acid | non-nephrotoxic | training | CC[C@H]1[C@@H](O)[C@H]2[C@@H]3CC[C@H]([C@H](C)CCC(O)=O)[C@@]3(C)CC[C@@H]2[C@@]2(C)CC[C@@H](O)C[C@@H]12 |
| Cefdinir | nephrotoxic | training | NC1=NC(=CS1)C(=N\O)\C(=O)N[C@H]1[C@H]2SCC(C=C)=C(N2C1=O)C(O)=O |
| Fludrocortisone | non-nephrotoxic | training | C[C@]12C[C@H](O)[C@@]3(F)[C@@H](CCC4=CC(=O)CC[C@]34C)[C@@H]1CC[C@]2(O)C(=O)CO |
| Stiripentol | non-nephrotoxic | training | CC(C)(C)C(O)C=CC1=CC=C2OCOC2=C1 |
| Caspofungin | nephrotoxic | training | CCC(C)CC(C)CCCCCCCCC(=O)N[C@H]1C[C@@H](O)[C@@H](NCCN)NC(=O)[C@@H]2[C@@H](O)CCN2C(=O)[C@@H](NC(=O)[C@@H](NC(=O)[C@@H]2C[C@@H](O)CN2C(=O)[C@@H](NC1=O)[C@@H](C)O)[C@H](O)[C@@H](O)C1=CC=C(O)C=C1)[C@H](O)CCN |
| Eplerenone | nephrotoxic | training | COC(=O)[C@@H]1CC2=CC(=O)CC[C@]2(C)[C@@]23O[C@@H]2C[C@@]2(C)[C@@H](CC[C@@]22CCC(=O)O2)[C@H]13 |
| sulfasalazine | nephrotoxic | training | OC(=O)C1=CC(=CC=C1O)N=NC1=CC=C(C=C1)S(=O)(=O)NC1=NC=CC=C1 |
| Ergoatetrine | non-nephrotoxic | training | CC(CO)NC(=O)C1CN(C)C2CC3=CNC4=CC=CC(=C34)C2=C1 |
| Fluvastatin | nephrotoxic | training | CC(C)N1C(\C=C\[C@@H](O)C[C@@H](O)CC(O)=O)=C(C2=CC=CC=C12)C1=CC=C(F)C=C1 |
| Trazodone | non-nephrotoxic | training | ClC1=CC(=CC=C1)N1CCN(CCCN2N=C3C=CC=CN3C2=O)CC1 |
| Bisacodyl | non-nephrotoxic | training | CC(=O)OC1=CC=C(C=C1)C(C1=CC=C(OC(C)=O)C=C1)C1=NC=CC=C1 |
| Raltegravir potassium | nephrotoxic | training | CN1C(=O)C([O-])=C(N=C1C(C)(C)NC(=O)C1=NN=C(C)O1)C(=O)NCC1=CC=C(F)C=C1 |
| Pinaverium bromide | non-nephrotoxic | training | COC1=CC(Br)=C(C[N+]2(CCOCCC3CCC4CC3C4(C)C)CCOCC2)C=C1OC |
| 2-[Bis[2-[bis(carboxylatomethyl)amino]ethyl]amino]acetate;gadolinium(3+) | nephrotoxic | training | [O-]C(=O)CN(CCN(CC([O-])=O)CC([O-])=O)CCN(CC([O-])=O)CC([O-])=O |
| Atenolol | nephrotoxic | training | CC(C)NCC(O)COC1=CC=C(CC(N)=O)C=C1 |
| Levofloxacin | nephrotoxic | training | C[C@H]1COC2=C(N3CCN(C)CC3)C(F)=CC3=C2N1C=C(C(O)=O)C3=O |
| Darunavir | nephrotoxic | training | CC(C)CN(C[C@@H](O)[C@H](CC1=CC=CC=C1)NC(=O)O[C@H]1CO[C@H]2OCC[C@@H]12)S(=O)(=O)C1=CC=C(N)C=C1 |
| Cefotaxime | nephrotoxic | training | CO\N=C(/C(=O)N[C@H]1[C@H]2SCC(COC(C)=O)=C(N2C1=O)C(O)=O)C1=CSC(N)=N1 |
| Telmisartan | nephrotoxic | training | CCCC1=NC2=C(C)C=C(C=C2N1CC1=CC=C(C=C1)C1=C(C=CC=C1)C(O)=O)C1=NC2=CC=CC=C2N1C |
| Butenafine | non-nephrotoxic | training | CN(CC1=CC=C(C=C1)C(C)(C)C)CC1=C2C=CC=CC2=CC=C1 |
| Zoledronic acid | nephrotoxic | training | OC(CN1C=CN=C1)(P(O)(O)=O)P(O)(O)=O |
| Dabigatran etexilate | non-nephrotoxic | training | CCCCCCOC(=O)N=C(N)C1=CC=C(NCC2=NC3=CC(=CC=C3N2C)C(=O)N(CCC(=O)OCC)C2=NC=CC=C2)C=C1 |
| Ciprofloxacin | nephrotoxic | training | OC(=O)C1=CN(C2CC2)C2=C(C=C(F)C(=C2)N2CCNCC2)C1=O |
| Propoxyphene | non-nephrotoxic | training | CCC(=O)O[C@@](CC1=CC=CC=C1)([C@H](C)CN(C)C)C1=CC=CC=C1 |
| Glatiramer | nephrotoxic | training | N[C@@H](CC1=CC=C(O)C=C1)C(O)=O |
| Neomycin | non-nephrotoxic | training | NC[C@@H]1O[C@H](O[C@@H]2[C@@H](CO)O[C@@H](O[C@@H]3[C@@H](O)[C@H](N)C[C@H](N)[C@H]3O[C@H]3O[C@H](CN)[C@@H](O)[C@H](O)[C@H]3N)[C@@H]2O)[C@H](N)[C@@H](O)[C@@H]1O |
| Norepinephrine | non-nephrotoxic | training | NCC(O)C1=CC=C(O)C(O)=C1 |
| Rabeprazole | nephrotoxic | training | COCCCOC1=CC=NC(CS(=O)C2=NC3=CC=CC=C3N2)=C1C |
| Epitopic | non-nephrotoxic | training | CCCC(=O)OC1(CCC2C3CC(F)C4=CC(=O)C=CC4(C)C3(F)C(O)CC12C)C(=O)COC(C)=O |
| Chlorpropamide | non-nephrotoxic | training | CCCNC(=O)NS(=O)(=O)C1=CC=C(Cl)C=C1 |
| Spectracef | nephrotoxic | training | CO\N=C(/C(=O)N[C@H]1[C@H]2SCC(\C=C/C3=C(C)N=CS3)=C(N2C1=O)C(=O)OCOC(=O)C(C)(C)C)C1=CSC(N)=N1 |
| Benzocaine | non-nephrotoxic | training | CCOC(=O)C1=CC=C(N)C=C1 |
| Dexlansoprazole | nephrotoxic | training | CC1=C(C[S@@](=O)C2=NC3=CC=CC=C3N2)N=CC=C1OCC(F)(F)F |
| Efavirenz | nephrotoxic | training | FC(F)(F)[C@]1(OC(=O)NC2=C1C=C(Cl)C=C2)C#CC1CC1 |
| Aliskiren | nephrotoxic | training | COCCCOC1=CC(C[C@@H](C[C@H](N)[C@@H](O)C[C@@H](C(C)C)C(=O)NCC(C)(C)C(N)=O)C(C)C)=CC=C1OC |
| Molindone | non-nephrotoxic | training | CCC1=C(C)NC2=C1C(=O)C(CN1CCOCC1)CC2 |
| Paroxetine | nephrotoxic | training | FC1=CC=C(C=C1)[C@@H]1CCNC[C@H]1COC1=CC=C2OCOC2=C1 |
| Rifaximin | non-nephrotoxic | training | CO[C@H]1C=CO[C@@]2(C)OC3=C(C)C(O)=C4C(O)=C(NC(=O)C(C)=CC=C[C@H](C)[C@H](O)[C@@H](C)[C@@H](O)[C@@H](C)[C@H](OC(C)=O)[C@@H]1C)C1=C(N=C5C=C(C)C=CN15)C4=C3C2=O |
| Eltrombopag | non-nephrotoxic | training | CC1=C(N=NC2=C(O)C(=CC=C2)C2=CC=CC(=C2)C(O)=O)C(=O)N(N1)C1=CC=C(C)C(C)=C1 |
| Clevidipine | nephrotoxic | training | CCCC(=O)OCOC(=O)C1=C(C)NC(C)=C(C1C1=CC=CC(Cl)=C1Cl)C(=O)OC |
| Tipranavir disodium | nephrotoxic | training | CCC[C@@]1(CCC2=CC=CC=C2)CC([O-])=C([C@H](CC)C2=CC=CC([N-]S(=O)(=O)C3=CC=C(C=N3)C(F)(F)F)=C2)C(=O)O1 |
| Frovatriptan | nephrotoxic | training | CN[C@@H]1CCC2=C(C1)C1=CC(=CC=C1N2)C(N)=O |
| Alvimopan | non-nephrotoxic | training | C[C@H]1CN(C[C@H](CC2=CC=CC=C2)C(=O)NCC(O)=O)CC[C@@]1(C)C1=CC=CC(O)=C1 |
| Propranolol | non-nephrotoxic | training | CC(C)NCC(O)COC1=C2C=CC=CC2=CC=C1 |
| Suprax | nephrotoxic | training | NC1=NC(=CS1)C(=N/OCC(O)=O)\C(=O)N[C@H]1[C@H]2SCC(C=C)=C(N2C1=O)C(O)=O |
| Penicillin G procaine | non-nephrotoxic | training | CC1(C)S[C@@H]2[C@H](NC(=O)CC3=CC=CC=C3)C(=O)N2[C@H]1C(O)=O |
| Calcipotriol hydrate | non-nephrotoxic | training | CC(C=CC(O)C1CC1)C1CCC2C(CCCC12C)=CC=C1CC(O)CC(O)C1=C |
| Isosorbide mononitrate | non-nephrotoxic | training | O[C@H]1CO[C@@H]2[C@@H](CO[C@H]12)O[N+]([O-])=O |
| Temsirolimus | nephrotoxic | training | CO[C@@H]1C[C@H](C[C@@H](C)[C@@H]2CC(=O)[C@H](C)\C=C(C)/[C@@H](O)[C@@H](OC)C(=O)[C@H](C)C[C@H](C)\C=C\C=C\C=C(C)\[C@H](C[C@@H]3CC[C@@H](C)[C@@](O)(O3)C(=O)C(=O)N3CCCC[C@H]3C(=O)O2)OC)CC[C@H]1OC(=O)C(C)(CO)CO |
| Paricalcitol | nephrotoxic | training | C[C@H](\C=C\[C@H](C)C(C)(C)O)[C@H]1CC[C@H]2\C(CCC[C@]12C)=C\C=C1C[C@@H](O)C[C@H](O)C1 |
| Zolmitriptan | nephrotoxic | training | CN(C)CCC1=CNC2=CC=C(C[C@H]3COC(=O)N3)C=C12 |
| Methylnaltrexone bromide | non-nephrotoxic | training | C[N+]1(CC2CC2)CC[C@@]23[C@H]4OC5=C2C(C[C@@H]1[C@]3(O)CCC4=O)=CC=C5O |
| Promethazine | non-nephrotoxic | training | CC(CN1C2=C(SC3=C1C=CC=C3)C=CC=C2)N(C)C |
| Febuxostat | nephrotoxic | training | CC(C)COC1=CC=C(C=C1C#N)C1=NC(C)=C(S1)C(O)=O |
| Lenalidomide | nephrotoxic | training | NC1=CC=CC2=C1CN(C1CCC(=O)NC1=O)C2=O |
| Zidovudine | nephrotoxic | training | CC1=CN([C@H]2C[C@H](N=[N+]=[N-])[C@@H](CO)O2)C(=O)NC1=O |
| Octreotide (acetate) | nephrotoxic | training | CC(O)C(CO)NC(=O)C1CSSCC(NC(=O)C(N)CC2=CC=CC=C2)C(=O)NC(CC2=CC=CC=C2)C(=O)NC(CC2=CNC3=CC=CC=C23)C(=O)NC(CCCCN)C(=O)NC(C(C)O)C(=O)N1 |
| Carvedilol | nephrotoxic | training | COC1=C(OCCNCC(O)COC2=CC=CC3=C2C2=C(N3)C=CC=C2)C=CC=C1 |
| Zolpidem | nephrotoxic | training | CN(C)C(=O)CC1=C(N=C2C=CC(C)=CN12)C1=CC=C(C)C=C1 |
| Deferoxamine | nephrotoxic | training | CC(=O)N(O)CCCCCNC(=O)CCC(=O)N(O)CCCCCNC(=O)CCC(=O)N(O)CCCCCN |
| Mesalamine | nephrotoxic | training | NC1=CC=C(O)C(=C1)C(O)=O |
| Gabapentin | nephrotoxic | training | NCC1(CC(O)=O)CCCCC1 |
| Guanfacine | nephrotoxic | training | NC(N)=NC(=O)CC1=C(Cl)C=CC=C1Cl |
| Vigabatrin | nephrotoxic | training | NC(CCC(O)=O)C=C |
| Memantine | nephrotoxic | training | CC12CC3CC(C)(C1)CC(N)(C3)C2 |
| Ropinirole | nephrotoxic | training | CCCN(CCC)CCC1=CC=CC2=C1CC(=O)N2 |
| Fenofibrate | nephrotoxic | training | CC(C)OC(=O)C(C)(C)OC1=CC=C(C=C1)C(=O)C1=CC=C(Cl)C=C1 |
| (-)-Chloramphenicol | nephrotoxic | training | OCC(NC(=O)C(Cl)Cl)C(O)C1=CC=C(C=C1)[N+]([O-])=O |
| Pimecrolimus | non-nephrotoxic | training | CC[C@@H]1C=C(C)C[C@H](C)C[C@H](OC)[C@H]2O[C@](O)([C@H](C)C[C@@H]2OC)C(=O)C(=O)N2CCCC[C@H]2C(=O)O[C@@H]([C@H](C)[C@@H](O)CC1=O)C(C)=C[C@@H]1CC[C@H](Cl)[C@@H](C1)OC |
| Bendroflumethiazide | non-nephrotoxic | training | NS(=O)(=O)C1=CC2=C(NC(CC3=CC=CC=C3)NS2(=O)=O)C=C1C(F)(F)F |
| Benzydamine | non-nephrotoxic | training | CN(C)CCCOC1=NN(CC2=CC=CC=C2)C2=CC=CC=C12 |
| Xylosyladenine | non-nephrotoxic | training | NC1=C2N=CN(C3OC(CO)C(O)C3O)C2=NC=N1 |
| Fesoterodine | non-nephrotoxic | training | CC(C)N(CC[C@H](C1=CC=CC=C1)C1=CC(CO)=CC=C1OC(=O)C(C)C)C(C)C |
| Colestipol | non-nephrotoxic | training | NCCNCCNCCNCCN |
| Benzoyl peroxide | non-nephrotoxic | training | O=C(OOC(=O)C1=CC=CC=C1)C1=CC=CC=C1 |
| Etomidate | non-nephrotoxic | training | CCOC(=O)C1=CN=CN1C(C)C1=CC=CC=C1 |
| Dobutamine | non-nephrotoxic | training | CC(CCC1=CC=C(O)C=C1)NCCC1=CC=C(O)C(O)=C1 |
| 6a-beta-Aporphine-10,11-diol | non-nephrotoxic | training | CN1CCC2=CC=CC3=C2C1CC1=C3C(O)=C(O)C=C1 |
| Lidocaine | non-nephrotoxic | training | CCN(CC)CC(=O)NC1=C(C)C=CC=C1C |
| Minocycline(1-) | nephrotoxic | validation | CN(C)C1=CC=C(O)C2=C1C[C@H]1C[C@H]3[C@H]([NH+](C)C)C(=O)C(C(N)=O)=C([O-])[C@@]3(O)C(=O)C1=C2[O-] |
| Sertraline hydrochloride | nephrotoxic | validation | CN[C@H]1CC[C@@H](C2=CC=C(Cl)C(Cl)=C2)C2=C1C=CC=C2 |
| Ketorolac | nephrotoxic | validation | OC(=O)C1CCN2C1=CC=C2C(=O)C1=CC=CC=C1 |
| Vancomycin | nephrotoxic | validation | CN[C@H](CC(C)C)C(=O)N[C@@H]1[C@H](O)C2=CC=C(OC3=C(O[C@@H]4O[C@H](CO)[C@@H](O)[C@H](O)[C@H]4O[C@H]4C[C@](C)(N)[C@H](O)[C@H](C)O4)C4=CC(=C3)[C@@H](NC(=O)[C@H](CC(N)=O)NC1=O)C(=O)N[C@@H]1C3=CC=C(O)C(=C3)C3=C(C=C(O)C=C3O)[C@H](NC(=O)[C@@H](NC1=O)[C@H](O)C1=CC(Cl)=C(O4)C=C1)C(O)=O)C(Cl)=C2 |
| Theophylline | nephrotoxic | validation | CN1C2=C(NC=N2)C(=O)N(C)C1=O |
| Tramadol | nephrotoxic | validation | COC1=CC(=CC=C1)[C@@]1(O)CCCC[C@@H]1CN(C)C |
| Sorafenib | nephrotoxic | validation | CNC(=O)C1=CC(OC2=CC=C(NC(=O)NC3=CC=C(Cl)C(=C3)C(F)(F)F)C=C2)=CC=N1 |
| Telbivudine | nephrotoxic | validation | CC1=CN([C@@H]2C[C@@H](O)[C@H](CO)O2)C(=O)NC1=O |
| Cetirizine | nephrotoxic | validation | OC(=O)COCCN1CCN(CC1)C(C1=CC=CC=C1)C1=CC=C(Cl)C=C1 |
| Ribavirin | nephrotoxic | validation | NC(=O)C1=NN(C=N1)[C@@H]1O[C@H](CO)[C@@H](O)[C@H]1O |
| CID 4673 | nephrotoxic | validation | NCCC(O)(P(O)([O-])=O)P(O)([O-])=O |
| Amphotericin b | nephrotoxic | validation | C[C@H]1O[C@@H](O[C@@H]2C[C@@H]3O[C@@](O)(C[C@H](O)[C@H]3C(O)=O)C[C@@H](O)C[C@@H](O)[C@H](O)CC[C@@H](O)C[C@@H](O)CC(=O)O[C@@H](C)[C@H](C)[C@H](O)[C@@H](C)\C=C\C=C\C=C\C=C\C=C\C=C\C=C\2)[C@@H](O)[C@@H](N)[C@@H]1O |
| Nabumetone | nephrotoxic | validation | COC1=CC2=CC=C(CCC(C)=O)C=C2C=C1 |
| Amprenavir | nephrotoxic | validation | CC(C)CN(C[C@@H](O)[C@H](CC1=CC=CC=C1)NC(=O)O[C@H]1CCOC1)S(=O)(=O)C1=CC=C(N)C=C1 |
| Erlotinib | nephrotoxic | validation | COCCOC1=C(OCCOC)C=C2C(NC3=CC(=CC=C3)C#C)=NC=NC2=C1 |
| Empagliflozin | nephrotoxic | validation | OC[C@H]1O[C@H]([C@H](O)[C@@H](O)[C@@H]1O)C1=CC=C(Cl)C(CC2=CC=C(O[C@H]3CCOC3)C=C2)=C1 |
| Nilotinib | nephrotoxic | validation | CC1=CN(C=N1)C1=CC(NC(=O)C2=CC=C(C)C(NC3=NC(=CC=N3)C3=CC=CN=C3)=C2)=CC(=C1)C(F)(F)F |
| 4-Phenylbutyric acid | nephrotoxic | validation | OC(=O)CCCC1=CC=CC=C1 |
| Fluconazole | nephrotoxic | validation | OC(CN1C=NC=N1)(CN1C=NC=N1)C1=CC=C(F)C=C1F |
| CID 5362051 | nephrotoxic | validation | CC(C)(O\N=C(\C(=O)N[C@H]1[C@H]2SCC(C[N+]3=CC=CC=C3)=C(N2C1=O)C([O-])=O)C1=CSC(N)=N1)C(O)=O |
| 2'-Epi-Perindopril, (2'R)- | nephrotoxic | validation | CCC[C@H](N[C@H](C)C(=O)N1[C@H]2CCCC[C@H]2C[C@H]1C(O)=O)C(=O)OCC |
| Trandolaprilat | nephrotoxic | validation | C[C@H](N[C@@H](CCC1=CC=CC=C1)C(O)=O)C(=O)N1[C@H]2CCCC[C@@H]2C[C@H]1C(O)=O |
| CID 16157882 | nephrotoxic | validation | CC[C@H](C)[C@H](NC(=O)[C@H](CC1=CC=CC=C1)NC(=O)[C@H](CC(C)C)NC(=O)[C@H](CCCNC(N)=N)NC(=O)[C@@H](NC(=O)[C@H](C)NC(=O)[C@H](CCC(O)=O)NC(=O)[C@H](CCC(O)=O)NC(=O)[C@H](CCC(O)=O)NC(=O)[C@H](CCSC)NC(=O)[C@H](CCC(N)=O)NC(=O)[C@H](CCCCN)NC(=O)[C@H](CO)NC(=O)[C@H](CC(C)C)NC(=O)[C@H](CC(O)=O)NC(=O)[C@H](CO)NC(=O)[C@@H](NC(=O)[C@H](CC1=CC=CC=C1)NC(=O)[C@@H](NC(=O)CNC(=O)[C@H](CCC(O)=O)NC(=O)CNC(=O)[C@@H](N)CC1=CN=CN1)[C@@H](C)O)[C@@H](C)O)C(C)C)C(=O)N[C@@H](CCC(O)=O)C(=O)N[C@@H](CC1=CNC2=CC=CC=C12)C(=O)N[C@@H](CC(C)C)C(=O)N[C@@H](CCCCN)C(=O)N[C@@H](CC(N)=O)C(=O)NCC(=O)NCC(=O)N1CCC[C@H]1C(=O)N[C@@H](CO)C(=O)N[C@@H](CO)C(=O)NCC(=O)N[C@@H](C)C(=O)N1CCC[C@H]1C(=O)N1CCC[C@H]1C(=O)N1CCC[C@H]1C(=O)N[C@@H](CO)C(N)=O |
| Sodium;(Z)-(2-methyl-1,1,4-trioxo-1lambda6,2-benzothiazin-3-ylidene)-(pyridin-2-ylamino)methanolate | nephrotoxic | validation | CN1\C(=C(/[O-])NC2=NC=CC=C2)C(=O)C2=C(C=CC=C2)S1(=O)=O |
| Carfilzomib | nephrotoxic | validation | CC(C)C[C@H](NC(=O)[C@H](CCC1=CC=CC=C1)NC(=O)CN1CCOCC1)C(=O)N[C@@H](CC1=CC=CC=C1)C(=O)N[C@@H](CC(C)C)C(=O)[C@@]1(C)CO1 |
| Fosphenytoin sodium | nephrotoxic | validation | [O-]P([O-])(=O)OCN1C(=O)NC(C1=O)(C1=CC=CC=C1)C1=CC=CC=C1 |
| Liraglutide | nephrotoxic | validation | CCCCCCCCCCCCCCCC(=O)N[C@@H](CCC(=O)NCCCC[C@H](NC(=O)[C@H](C)NC(=O)[C@H](C)NC(=O)[C@H](CCC(N)=O)NC(=O)CNC(=O)[C@H](CCC(O)=O)NC(=O)[C@H](CC(C)C)NC(=O)[C@H](CC1=CC=C(O)C=C1)NC(=O)[C@H](CO)NC(=O)[C@H](CO)NC(=O)[C@@H](NC(=O)[C@H](CC(O)=O)NC(=O)[C@H](CO)NC(=O)[C@@H](NC(=O)[C@H](CC1=CC=CC=C1)NC(=O)[C@@H](NC(=O)CNC(=O)[C@H](CCC(O)=O)NC(=O)[C@H](C)NC(=O)[C@@H](N)CC1=CN=CN1)[C@@H](C)O)[C@@H](C)O)C(C)C)C(=O)N[C@@H](CCC(O)=O)C(=O)N[C@@H](CC1=CC=CC=C1)C(=O)N[C@@H]([C@@H](C)CC)C(=O)N[C@@H](C)C(=O)N[C@@H](CC1=CNC2=CC=CC=C12)C(=O)N[C@@H](CC(C)C)C(=O)N[C@@H](C(C)C)C(=O)N[C@@H](CCCNC(N)=N)C(=O)NCC(=O)N[C@@H](CCCNC(N)=N)C(=O)NCC(O)=O)C(O)=O |
| Eletriptan | nephrotoxic | validation | CN1CCC[C@@H]1CC1=CNC2=CC=C(CCS(=O)(=O)C3=CC=CC=C3)C=C12 |
| Nevirapine | nephrotoxic | validation | CC1=CC=NC2=C1NC(=O)C1=C(N=CC=C1)N2C1CC1 |
| Crizotinib | nephrotoxic | validation | C[C@@H](OC1=CC(=CN=C1N)C1=CN(N=C1)C1CCNCC1)C1=C(Cl)C(F)=CC=C1Cl |
| Delavirdine | nephrotoxic | validation | CC(C)NC1=CC=CN=C1N1CCN(CC1)C(=O)C1=CC2=CC(NS(C)(=O)=O)=CC=C2N1 |
| Zaleplon | nephrotoxic | validation | CCN(C(C)=O)C1=CC(=CC=C1)C1=CC=NC2=C(C=NN12)C#N |
| Perflutren | nephrotoxic | validation | FC(F)(F)C(F)(F)C(F)(F)F |
| Fungizone | nephrotoxic | validation | CC1OC(O[C@@H]2C[C@@H]3O[C@@](O)(C[C@H](O)[C@H]3C(O)=O)C[C@@H](O)C[C@@H](O)[C@H](O)CC[C@@H](O)C[C@@H](O)CC(=O)O[C@@H](C)[C@H](C)[C@H](O)[C@@H](C)\C=C\C=C\C=C\C=C\C=C\C=C\C=C\2)C(O)C(N)C1O |
| clozapine | nephrotoxic | validation | CN1CCN(CC1)C1=NC2=C(NC3=C1C=CC=C3)C=CC(Cl)=C2 |
| ganciclovir | nephrotoxic | validation | NC1=NC2=C(N=CN2COC(CO)CO)C(=O)N1 |
| Ramiprilat | nephrotoxic | validation | C[C@H](N[C@@H](CCC1=CC=CC=C1)C(O)=O)C(=O)N1[C@H]2CCC[C@H]2C[C@H]1C(O)=O |
| Citalopram | nephrotoxic | validation | CN(C)CCCC1(OCC2=C1C=CC(=C2)C#N)C1=CC=C(F)C=C1 |
| Rufinamide | nephrotoxic | validation | NC(=O)C1=CN(CC2=C(F)C=CC=C2F)N=N1 |
| Cilazapril monohydrate | nephrotoxic | validation | CCOC(=O)[C@H](CCC1=CC=CC=C1)N[C@H]1CCCN2CCC[C@H](N2C1=O)C(O)=O |
| Tenofovir | nephrotoxic | validation | C[C@H](CN1C=NC2=C(N)N=CN=C12)OCP(O)(O)=O |
| Fludarabine | nephrotoxic | validation | NC1=C2N=CN([C@@H]3O[C@H](CO)[C@@H](O)[C@@H]3O)C2=NC(F)=N1 |
| Moxifloxacin hydrochloride | nephrotoxic | validation | COC1=C(N2C[C@@H]3CCCN[C@@H]3C2)C(F)=CC2=C1N(C=C(C(O)=O)C2=O)C1CC1 |
| Strontium ranelate | non-nephrotoxic | validation | [O-]C(=O)CN(CC([O-])=O)C1=C(C#N)C(CC([O-])=O)=C(S1)C([O-])=O |
| (S)-Tamsulosin Hydrochloride | non-nephrotoxic | validation | CCOC1=C(OCCN[C@@H](C)CC2=CC=C(OC)C(=C2)S(N)(=O)=O)C=CC=C1 |
| Dofetilide | non-nephrotoxic | validation | CN(CCOC1=CC=C(NS(C)(=O)=O)C=C1)CCC1=CC=C(NS(C)(=O)=O)C=C1 |
| Imiquimod | non-nephrotoxic | validation | CC(C)CN1C=NC2=C1C1=CC=CC=C1N=C2N |
| Atosiban | non-nephrotoxic | validation | CCOC1=CC=C(C[C@H]2NC(=O)CCSSC[C@H](NC(=O)[C@H](CC(N)=O)NC(=O)C(NC(=O)[C@@H](NC2=O)[C@@H](C)CC)[C@@H](C)O)C(=O)N2CCC[C@H]2C(=O)N[C@@H](CCCN)C(=O)NCC(N)=O)C=C1 |
| Pravastatin | nephrotoxic | validation | CC[C@H](C)C(=O)O[C@H]1C[C@H](O)C=C2C=C[C@H](C)[C@H](CC[C@@H](O)C[C@@H](O)CC(O)=O)[C@@H]12 |
| Mycophenolate mofetil | nephrotoxic | validation | COC1=C(C)C2=C(C(=O)OC2)C(O)=C1C\C=C(/C)CCC(=O)OCCN1CCOCC1 |
| Fenofibric acid | nephrotoxic | validation | CC(C)(OC1=CC=C(C=C1)C(=O)C1=CC=C(Cl)C=C1)C(O)=O |
| Testosterone | nephrotoxic | validation | C[C@]12CC[C@H]3[C@@H](CCC4=CC(=O)CC[C@]34C)[C@@H]1CC[C@@H]2O |
| allopurinol | nephrotoxic | validation | O=C1NC=NC2=C1C=NN2 |
| (2-Azanidylcyclohexyl)azanide;oxalic acid;platinum(2+) | nephrotoxic | validation | [NH-]C1CCCCC1[NH-] |
| Etoricoxib | nephrotoxic | validation | CC1=CC=C(C=N1)C1=NC=C(Cl)C=C1C1=CC=C(C=C1)S(C)(=O)=O |
| Tizanidine | nephrotoxic | validation | ClC1=C(NC2=NCCN2)C2=NSN=C2C=C1 |
| Glutamine | nephrotoxic | validation | N[C@@H](CCC(N)=O)C(O)=O |
| Cyclosporine | nephrotoxic | validation | CCC1NC(=O)C(C(O)C(C)C\C=C\C)N(C)C(=O)C(C(C)C)N(C)C(=O)C(CC(C)C)N(C)C(=O)C(CC(C)C)N(C)C(=O)C(C)NC(=O)C(C)NC(=O)C(CC(C)C)N(C)C(=O)C(NC(=O)C(CC(C)C)N(C)C(=O)CN(C)C1=O)C(C)C |
| Etodolac | nephrotoxic | validation | CCC1=C2NC3=C(CCOC3(CC)CC(O)=O)C2=CC=C1 |
| Doxazosin | nephrotoxic | validation | COC1=C(OC)C=C2C(N)=NC(=NC2=C1)N1CCN(CC1)C(=O)C1COC2=C(O1)C=CC=C2 |
| Amorolfinehydrochloride | non-nephrotoxic | validation | CCC(C)(C)C1=CC=C(CC(C)CN2CC(C)OC(C)C2)C=C1 |
| Fluvean | non-nephrotoxic | validation | CC1(C)OC2CC3C4CC(F)C5=CC(=O)C=CC5(C)C4(F)C(O)CC3(C)C2(O1)C(=O)CO |
| Clobetasol | non-nephrotoxic | validation | CC1CC2C3CCC4=CC(=O)C=CC4(C)[C@@]3(F)C(O)CC2(C)[C@@]1(O)C(=O)CCl |
| Pioglitazone | non-nephrotoxic | validation | CCC1=CC=C(CCOC2=CC=C(CC3SC(=O)NC3=O)C=C2)N=C1 |
| Moxonidine | non-nephrotoxic | validation | COC1=NC(C)=NC(Cl)=C1NC1=NCCN1 |
| Prednicarbate | non-nephrotoxic | validation | CCOC(=O)OC1(CCC2C3CCC4=CC(=O)C=CC4(C)C3C(O)CC12C)C(=O)COC(=O)CC |
| Ondansetron | non-nephrotoxic | validation | CN1C2=C(C3=CC=CC=C13)C(=O)C(CN1C=CN=C1C)CC2 |
| Ergotamine | non-nephrotoxic | validation | CN1CC(C=C2C1CC1=CNC3=CC=CC2=C13)C(=O)NC1(C)OC2(O)C3CCCN3C(=O)C(CC3=CC=CC=C3)N2C1=O |
| Norgestrel | non-nephrotoxic | validation | CCC12CCC3C(CCC4=CC(=O)CCC34)C1CCC2(O)C#C |
| Methyltestosterone | non-nephrotoxic | validation | CC1(O)CCC2C3CCC4=CC(=O)CCC4(C)C3CCC12C |
| Lisuride | non-nephrotoxic | validation | CCN(CC)C(=O)NC1CN(C)C2CC3=CNC4=CC=CC(=C34)C2=C1 |
| Fernisone | non-nephrotoxic | validation | CC12CC(=O)C3C(CCC4=CC(=O)C=CC34C)C1CCC2(O)C(=O)CO |
| Ambrisentan | non-nephrotoxic | validation | COC(C(OC1=NC(C)=CC(C)=N1)C(O)=O)(C1=CC=CC=C1)C1=CC=CC=C1 |
| Methergin (Salt/Mix) | non-nephrotoxic | validation | CCC(CO)NC(=O)C1CN(C)C2CC3=CNC4=CC=CC(=C34)C2=C1 |
| Naftifine | non-nephrotoxic | validation | CN(CC=CC1=CC=CC=C1)CC1=C2C=CC=CC2=CC=C1 |
| BiDil (Salt/Mix) | non-nephrotoxic | validation | [O-][N+](=O)OC1COC2C(COC12)O[N+]([O-])=O |
| Nateglinide | non-nephrotoxic | validation | CC(C)C1CCC(CC1)C(=O)NC(CC1=CC=CC=C1)C(O)=O |
| Hydroquinone | non-nephrotoxic | validation | OC1=CC=C(O)C=C1 |
| Diltiazem | non-nephrotoxic | validation | COC1=CC=C(C=C1)C1SC2=C(C=CC=C2)N(CC[NH+](C)C)C(=O)C1OC(C)=O |
| Econazole | non-nephrotoxic | validation | ClC1=CC=C(COC(CN2C=CN=C2)C2=CC=C(Cl)C=C2Cl)C=C1 |
| Flavoxate | non-nephrotoxic | validation | CC1=C(OC2=C(C=CC=C2C(=O)OCCN2CCCCC2)C1=O)C1=CC=CC=C1 |
| Ciclopirox | non-nephrotoxic | validation | CC1=CC(=O)N(O)C(=C1)C1CCCCC1 |
| Methoxsalen | non-nephrotoxic | validation | COC1=C2OC=CC2=CC2=C1OC(=O)C=C2 |
| Pamine | non-nephrotoxic | validation | C[N+]1(C)C2CC(CC1C1OC21)OC(=O)C(CO)C1=CC=CC=C1 |
| Cortisone | non-nephrotoxic | validation | C[C@]12CC(=O)[C@H]3[C@@H](CCC4=CC(=O)CC[C@]34C)[C@@H]1CC[C@]2(O)C(=O)CO |
| Papaverine | non-nephrotoxic | validation | COC1=CC=C(CC2=C3C=C(OC)C(OC)=CC3=CC=N2)C=C1OC |
| Misoprostol | non-nephrotoxic | validation | CCCCC(C)(O)CC=C[C@H]1[C@H](O)CC(=O)[C@@H]1CCCCCCC(=O)OC |
| Verdeso | non-nephrotoxic | validation | CC1(C)O[C@@H]2C[C@H]3C4CCC5=CC(=O)C=CC5(C)[C@H]4C(O)CC3(C)[C@@]2(O1)C(=O)CO |
| Folliculinum | non-nephrotoxic | validation | CC12CCC3C(CCC4=C3C=CC(O)=C4)C1CCC2=O |
| Etonogestrel | non-nephrotoxic | validation | CCC12CC(=C)C3C(CCC4=CC(=O)CCC34)C1CC[C@@]2(O)C#C |
| Tromethamine | non-nephrotoxic | validation | NC(CO)(CO)CO |
| Azelaic acid | non-nephrotoxic | validation | OC(=O)CCCCCCCC(O)=O |
| Glycopyrrolate | non-nephrotoxic | validation | C[N+]1(C)CCC(C1)OC(=O)C(O)(C1CCCC1)C1=CC=CC=C1 |
| Carbenicillin | non-nephrotoxic | validation | CC1(C)SC2C(NC(=O)C(C([O-])=O)C3=CC=CC=C3)C(=O)N2C1C([O-])=O |
| Lincocin | non-nephrotoxic | validation | CCCC1CC(N(C)C1)C(=O)NC(C(C)O)C1OC(SC)C(O)C(O)C1O |
| Sulfanilamide | non-nephrotoxic | validation | NC1=CC=C(C=C1)S(N)(=O)=O |
| Darifenacin | non-nephrotoxic | validation | NC(=O)C(C1CCN(CCC2=CC=C3OCCC3=C2)C1)(C1=CC=CC=C1)C1=CC=CC=C1 |
| Iron sucrose | non-nephrotoxic | validation | OC[C@H]1O[C@@](CO)(O[C@H]2O[C@H](CO)[C@@H](O)[C@H](O)[C@H]2O)[C@@H](O)[C@@H]1O |
| DL-Glutamine | non-nephrotoxic | validation | NC(CCC(N)=O)C(O)=O |
| Lanoxin | non-nephrotoxic | validation | CC1OC(CC(O)C1O)OC1C(O)CC(OC2C(O)CC(OC3CCC4(C)C(CCC5C4CC(O)C4(C)C(CCC54O)C4=CC(=O)OC4)C3)OC2C)OC1C |
| Antibiotic squibb 26 | non-nephrotoxic | validation | CC1C(NC(=O)C(=NOC(C)(C)C(O)=O)C2=CSC(N)=N2)C(=O)N1S(O)(=O)=O |
| Racemic-Timolol | non-nephrotoxic | validation | CC(C)(C)NCC(O)COC1=NSN=C1N1CCOCC1 |
| 16-Epiestriol | non-nephrotoxic | validation | CC12CCC3C(CCC4=C3C=CC(O)=C4)C1CC(O)C2O |
| Megestrol | non-nephrotoxic | validation | CC(=O)[C@@]1(O)CC[C@H]2[C@@H]3C=C(C)C4=CC(=O)CC[C@]4(C)[C@H]3CC[C@]12C |
| Penticort | non-nephrotoxic | validation | CC(=O)OCC(=O)C12OC3(CCCC3)OC1CC1C3CCC4=CC(=O)C=CC4(C)C3(F)C(O)CC21C |
| Retapamulin | non-nephrotoxic | validation | C[C@@H]1CC[C@@]23CCC(=O)[C@H]2[C@]1(C)[C@@H](C[C@@](C)(C=C)[C@@H](O)[C@@H]3C)OC(=O)CSC1C[C@@H]2CC[C@H](C1)N2C |
| Oxybutynin | non-nephrotoxic | validation | CCN(CC)CC#CCOC(=O)C(O)(C1CCCCC1)C1=CC=CC=C1 |
| Glycine | non-nephrotoxic | validation | NCC(O)=O |
| Carbimazole | non-nephrotoxic | validation | CCOC(=O)N1C=CN(C)C1=S |

Table S2

| **Bit** | **SMARTS** | **positive** | **negative** | **total** | **positive rate** |
| --- | --- | --- | --- | --- | --- |
| KR93 | [!#1][CH]([CH3])[NH]C(=O)[!#1] | 7 | 2 | 9 | 0.78 |
| KR98 | [!#1][CH]([CH3])C(=O)[!#1] | 24 | 7 | 31 | 0.77 |
| KR207 | [!#1][CH]1[CH2][CH2][CH]([!#1])[CH]([!#1])[CH2]1 | 5 | 1 | 6 | 0.83 |
| KR212 | [!#1][CH]1[CH2][CH2][CH2][CH2][CH]1[!#1] | 7 | 1 | 8 | 0.88 |
| KR218 | [!#1][CH]1[CH2][CH2][CH2][CH2]N1[!#1] | 5 | 1 | 6 | 0.83 |
| KR228 | [!#1][CH]1[CH2][CH2]1 | 10 | 3 | 13 | 0.77 |
| KR413 | [!#1][CH2][CH2]c1[cH][cH][cH][cH][cH]1 | 9 | 0 | 9 | 1.00 |
| KR476 | [!#1][CH2][NH][CH2][!#1] | 15 | 3 | 18 | 0.83 |
| KR599 | [!#1][CH2]c1[cH][nH]c2[cH][cH][cH][cH]c12 | 6 | 1 | 7 | 0.86 |
| KR663 | [!#1][CH2]S(=O)(=O)[!#1] | 5 | 1 | 6 | 0.83 |
| KR700 | [!#1][NH][CH]([CH2]c1[cH][nH]c2[cH][cH][cH][cH]c12)C(=O)[!#1] | 6 | 1 | 7 | 0.86 |
| KR707 | [!#1][NH][CH]([CH3])C(=O)[!#1] | 14 | 1 | 15 | 0.93 |
| KR842 | [!#1][NH]C(=O)[CH]([!#1])[CH3] | 7 | 1 | 8 | 0.88 |
| KR848 | [!#1][NH]C(=O)[CH]([CH3])[NH]C(=O)[!#1] | 7 | 0 | 7 | 1.00 |
| KR924 | [!#1][NH]C(=O)O[!#1] | 12 | 2 | 14 | 0.86 |
| KR1418 | [!#1]C(=O)C(=O)[!#1] | 9 | 1 | 10 | 0.90 |
| KR1536 | [!#1]C(F)(F)F | 14 | 3 | 17 | 0.82 |
| KR1798 | [!#1]c1[cH][cH]c(F)[cH][cH]1 | 8 | 0 | 8 | 1.00 |
| KR1920 | [!#1]c1[cH][nH]c2[cH][cH][cH][cH]c12 | 6 | 1 | 7 | 0.86 |
| KR2034 | [!#1]c1[cH]sc([!#1])n1 | 13 | 1 | 14 | 0.93 |
| KR2035 | [!#1]c1[cH]sc([NH2])n1 | 11 | 1 | 12 | 0.92 |
| KR2135 | [!#1]c1n[cH]n([!#1])c1[!#1] | 15 | 3 | 18 | 0.83 |
| KR2137 | [!#1]c1n[cH]nc([!#1])c1[!#1] | 7 | 2 | 9 | 0.78 |
| KR2149 | [!#1]c1nc([!#1])c([!#1])[nH]1 | 7 | 1 | 8 | 0.88 |
| KR2150 | [!#1]c1nc([!#1])c([!#1])c([!#1])n1 | 10 | 2 | 12 | 0.83 |
| KR2308 | [!#1]N([CH3])C(=O)[!#1] | 11 | 1 | 12 | 0.92 |
| KR2428 | [!#1]N1[CH2][CH2]N([!#1])[CH2][CH2]1 | 15 | 4 | 19 | 0.79 |
| KR2444 | [!#1]N1[CH2][CH2]N([CH3])[CH2][CH2]1 | 6 | 0 | 6 | 1.00 |
| KR2988 | C=C(NC=O)C=O | 13 | 1 | 14 | 0.93 |
| KR3145 | c1ccc2ncncc2c1 | 5 | 1 | 6 | 0.83 |
| KR3179 | C1CNCCN1 | 23 | 6 | 29 | 0.79 |
| KR3182 | c1cnoc1 | 5 | 1 | 6 | 0.83 |
| KR3200 | c1cscn1 | 18 | 2 | 20 | 0.90 |
| KR3206 | c1nc2ccccc2[nH]1 | 7 | 0 | 7 | 1.00 |
| KR3280 | CC(=O)c1ccc(N)cc1 | 8 | 0 | 8 | 1.00 |
| KR3403 | CC(C)N(C=O)C(C)C | 6 | 1 | 7 | 0.86 |
| KR3405 | CC(C)NCC(=O)N | 8 | 2 | 10 | 0.80 |
| KR3409 | CC(C)OC(=O)N | 7 | 1 | 8 | 0.88 |
| KR3415 | CC(N)c1ccccc1 | 16 | 4 | 20 | 0.80 |
| KR3419 | CC(NC=O)C(=O)O | 25 | 8 | 33 | 0.76 |
| KR3443 | CC=N | 22 | 7 | 29 | 0.76 |
| KR3452 | CC=NO | 13 | 1 | 14 | 0.93 |
| KR3540 | Cc1ccc(cc1)c2ccccc2 | 7 | 0 | 7 | 1.00 |
| KR3548 | Cc1ccc(F)cc1 | 8 | 0 | 8 | 1.00 |
| KR3586 | Cc1cccc(F)c1 | 12 | 0 | 12 | 1.00 |
| KR3598 | Cc1ccccc1C=O | 9 | 2 | 11 | 0.82 |
| KR3602 | Cc1ccccc1N | 27 | 7 | 34 | 0.79 |
| KR3625 | Cc1cscn1 | 14 | 2 | 16 | 0.88 |
| KR3767 | CCN(CCO)C=O | 34 | 10 | 44 | 0.77 |
| KR3792 | CCOC(=O)C(C)NC(=O)C | 7 | 1 | 8 | 0.88 |
| KR3793 | CCOC(=O)C(C)NC=O | 8 | 1 | 9 | 0.89 |
| KR3800 | CCOC(=O)CN | 14 | 1 | 15 | 0.93 |
| KR3801 | CCOC(=O)CNC=O | 8 | 1 | 9 | 0.89 |
| KR3901 | CN(C)S(=O)=O | 5 | 1 | 6 | 0.83 |
| KR3930 | CNC(=O)CCC(=O)O | 6 | 1 | 7 | 0.86 |
| KR3934 | CNC(=O)O | 14 | 3 | 17 | 0.82 |
| KR3938 | CNC(C)C(=O)O | 25 | 8 | 33 | 0.76 |
| KR3961 | COC(=O)C(C)NC=O | 9 | 1 | 10 | 0.90 |
| KR3966 | COC(=O)CN | 18 | 1 | 19 | 0.95 |
| KR4021 | CS(=O)(=O)N | 6 | 1 | 7 | 0.86 |
| KR4023 | CS(=O)=O | 9 | 2 | 11 | 0.82 |
| KR4029 | CS(c1nc2ccccc2[nH]1) | 6 | 0 | 6 | 1.00 |
| KR4053 | FC(F)F | 14 | 3 | 17 | 0.82 |
| KR4064 | Fc1cccc(C=O)c1 | 8 | 0 | 8 | 1.00 |
| KR4065 | Fc1cccc(F)c1 | 8 | 0 | 8 | 1.00 |
| KR4067 | Fc1ccccc1 | 29 | 1 | 30 | 0.97 |
| KR4081 | N#Cc1ccccc1 | 6 | 0 | 6 | 1.00 |
| KR4183 | NC(=O)CCC(=O)O | 6 | 1 | 7 | 0.86 |
| KR4188 | NC(=O)CCCO | 11 | 3 | 14 | 0.79 |
| KR4252 | Nc1ccc(F)cc1 | 11 | 0 | 11 | 1.00 |
| KR4274 | Nc1ncccn1 | 12 | 3 | 15 | 0.80 |
| KR4275 | Nc1nccs1 | 14 | 1 | 15 | 0.93 |
| KR4290 | NCCC(=O)O | 11 | 3 | 14 | 0.79 |
| KR4292 | NCCCC(=O)O | 13 | 3 | 16 | 0.81 |
| KR4472 | O=CC=O | 9 | 1 | 10 | 0.90 |
| KR4512 | O=CCC=O | 10 | 3 | 13 | 0.77 |
| KR4520 | O=CCCc1c[nH]c2ccccc12 | 6 | 1 | 7 | 0.86 |
| KR4523 | O=CCCCc1ccccc1 | 15 | 3 | 18 | 0.83 |
| KR4539 | O=CN1CCCCC1 | 8 | 1 | 9 | 0.89 |
| KR4556 | O=CNCCCCNC=O | 8 | 0 | 8 | 1.00 |
| KR4651 | OC(=O)C1CCCN1 | 8 | 0 | 8 | 1.00 |
| KR4669 | OC(=O)CCCCCNC=O | 6 | 1 | 7 | 0.86 |
| KR4671 | OC(=O)CCCNC=O | 7 | 2 | 9 | 0.78 |
| KR4674 | OC(=O)CCNC=O | 9 | 2 | 11 | 0.82 |
| KR4678 | OC(=O)CNC=O | 40 | 10 | 50 | 0.80 |
| KR4747 | Oc1cccc(C=O)c1 | 7 | 2 | 9 | 0.78 |
| KR4834 | ON=C | 13 | 1 | 14 | 0.93 |
